# Application of the effective diameters of porous media to the non-Darcy flow analyses

**DOI:** 10.1038/s41598-022-08135-x

**Published:** 2022-03-29

**Authors:** Chang-Hoon Shin

**Affiliations:** Research Institute, Korea Gas Corporation (KOGAS), Ansan, 15328 Gyeonggi-do South Korea

**Keywords:** Chemistry, Energy science and technology, Engineering, Materials science, Nanoscience and technology, Physics

## Abstract

Non-Darcy flows are a vital event leading to the inaccuracy in flow performance evaluation, particularly in the fractured wells in shale gas and tight oil deposits. The internal flow commonly indicates high flow rates at the early period of production, owing to the large geometric size of hydraulic fracture and high porosity in proppant packs. After that, the production flow rate decays steeply until the Darcy flow regime is attained. Therefore, accurate porous flow analyses from the Darcy to Forchheimer and then turbulent flow regimes are essential for optimal facility designs and economic productions in the unconventional deposits. The Comiti capillary model is recognised as the leading non-Darcy flow correlation for simple packed beds comprising mono-size grains. However, in actual hydraulic fractures, various types of proppants are used simultaneously and generally combined with numerous soil grains of different sizes and shapes. In this study, the Comiti model is modified by incorporating the effective diameters of the porous media and then examined for mixed complex multi-size packs. Subsequently, a novel type of non-Darcy flow equation is presented according to the logarithmic turbulent friction factor based on the physical variables determined only in the Darcy flow regime. The new equation presents accurate results for all the types of proppant packs under wide porosity and permeability ranges. The generalised non-Darcy flow correlation, which can be extensively employed from the Darcy to non-Darcy flow analyses, particularly beyond the Forchheimer regime, is presented for the accurate flow evaluation of the fractured reservoirs.

## Introduction

Non-Darcy flow is one of the most important issues affecting the productivity of hydraulically fractured reservoirs, such as shale gas, tight oil and geothermal water formations^1–5^. The internal flow through the hydraulic fracture comprising large numbers of micro-proppants and soil grains commonly indicates high flow rates in turbulence at the early period of production owing to the large geometric size of hydraulic fracture and high porosity in proppant packs. However, the flow rate reduces rapidly along with a decrease in formation pressure during fluid production in the early months or years and then declines slowly after attaining the Darcy flow via the Forchheimer regime^3–5^. Therefore, the dramatic flow changes are critical for economic production and optimal developments in unconventional oil and gas reservoirs^3–5^.

With an increase in the flow rate, inertial forces become increasingly substantial and the relationship between pressure gradient and seepage velocity becomes non-linear (weak inertia)^6–10^. With further increase, the pressure loss shifts from a weak to a strong (Forchheimer) inertia system, where the pressure drop is proportional to the square of the seepage velocity^6–10^. Forchheimer proposed the first equation of motion to account for non-linear effects, and several researchers have established correlations for the inertia resistance factor, often known as the beta factor (β), a parameter in the Forchheimer equation for quantifying the non-Darcy flow effect^11–15^. Ruth and Ma^16^ demonstrated that permeability is velocity-dependent and Forchheimer effects require a thorough understanding of the microscopic flow field, including the physical structure of the porous media as well as the flow patterns in different flow regimes.

The inertia resistance factor (β) can now be measured for proppant packs via laboratory experiments. Nevertheless, the determination of the β for actual formations, which normally have huge, complex and heterogeneous structures, is still a point of contention^2,17,18^. In addition to being controversial, bilinear behaviour in the Forchheimer graph at large Reynolds numbers has been widely reported in the petroleum-related literature for proppant packs^1–5,19^. The flow regime beyond the Forchheimer regime is highly implicated for flow in proppant packs practical flow rates interests^1–5,17^. Accurate analyses for both the non-Darcy flows, not only the Forchheimer regime but also the beyond Forchheimer regime, are crucial for optimal facility designs and economic production operations. Therefore, new correlations that can be extensively applied to both the non-Darcy flows without determining the Forchheimer coefficient should be presented for the accurate and extensive non-Darcy flow analyses.

## Concerns and limitations of the current non-Darcy flow analyses

The three key concerns in the non-Darcy flow features, i.e. the validity range of Darcy’s law, physical meaning of nonlinearity in the Forchheimer equation and generalised porous flow relationships for flow velocity changes, have received attention over the decades, resulting in numerous experimental and theoretical research^10,20^. The transition from the Darcy to the Forchheimer regime occurs in the range of *Re* = 1–15^9,16,21^. The numerical findings of Fourar et al.^22^ showed that the transition occurs around *Re*_*part*_ = 2–4. The onset of inertia flow was demonstrated by Garrouch and Ali^23^ using capillary pressure calculation and polymer flooding tests with sandstone cores. They suggested using the Forchheimer number instead of the Reynolds number to optimally predict the onset of inertia flow. In addition, Barrere^7^ and later Mei and Auriault^8^ described the weak inertia equation according to two-dimensional flow analyses. However, Fourar et al.^22^ confirmed that the transition domain is quite slim in three-dimensional flow cases and can be ignored for practical purposes^22^. Seguin et al.^24,25^ used electrochemical micro-probing technique to determine the limit of the Forchheimer regime and the onset of the fully established turbulent flow regime for packed beds with spheres and plates as well as for synthetic foams. The critical Reynolds number for the turbulent flow regime was reported in the range of *Re*_*part*_ = 80–475^24,25^. Critical Reynolds numbers of each flow regime can vary by an order of magnitude according to the individual pore structures of each medium; thus, no universal criteria for identifying flow regime shifts have been proposed yet. Therefore, new correlations that can reasonably analyse dramatic changes due to the flow regime variations should be deduced without requiring any criteria for the altered flow regimes.

The occurrence of turbulence was important in early descriptions of the physical mechanisms of the non-Darcy flow. The experimental results reported by Dybbs and Edwards^26^ started an argument that the deviations from Darcy’s law are not initiated by turbulence and such deviations do not essentially correspond to a different flow regime^15,20^. These authors also proposed the existence of four flow regimes: (1) Darcy (creeping flow) regime, which is dominated by viscous forces, (2) inertial flow regime, which begins where the boundary layers become more pronounced, (3) unsteady laminar flow regime, which is characterised by the event of wake oscillations and the development of vortices, and (4) highly unsteady and chaotic flow regime, which qualitatively mirrors the turbulent flow in pipes and is dominated by eddies. Therefore, Dybbs and Edwards^26^ insisted that all the flow regimes from (1) to (3) were classified as the types of laminar flows. It is now generally agreed that the quadratic term in the Forchheimer equation is associated with the inertia effect in the laminar regime and is fundamentally different from the quadratic velocity dependence for turbulent flow beyond the Forchheimer regime^17^ regardless of diverse opinions on the origin of the nonlinearity^15,20^. Therefore, the fractured reservoirs, mostly showing both the non-Darcy flows, cannot be accurately analysed using only the Forchheimer equation; thus, a new correlation applicable for both the non-Darcy flow regimes, particularly beyond the Forchheimer regime, should be invented.

Furthermore, the deviation of Darcy’s law or the Forchheimer equation has attracted attention in the recently developed artificial porous materials used for micro- and nano-thermal devices, biomaterials and chemistry and energy facilities^27–33^. Increasing the heat transfer rate with a reduction in the cost and size is a critical issue in several engineering applications^28^. Liquid–vapour phase change processes within porous media occur in numerous applications, where they are often driven by a complex interaction of gravitational, capillary and viscous forces^29^. The anisotropy of permeability and thermal conductivity considerably effects on the initiation and termination of the phase change process and heat transfer rate compared with those under isotropic conditions^30^. In a thermo-dynamical system, irreversibility occurs owing to the contribution of various factors such as resistance to the fluid flow, Joule heating, molecular vibration, diffusion, heat transport, chemical reaction and thermal radiation^31,32^. The Darcy–Forchheimer nano-liquid flow with entropy generation is essential for carbon nano-tubes owing to their various applications in heat exchangers, thermal power plants and microelectronics^33^.

Theoretically, derivation of Darcy’s law or the Forchheimer equation can be classified into macroscale and microscale approaches in terms of generalised porous flow relationships, depending on the starting point^34^. Macroscale approaches explore the origin of linearity or nonlinearity by recovering Darcy’s law or the Forchheimer equation from the Navier–Stokes equation at the continuum scale. However, microscale approaches use simple conceptual models as microscale representations for the frictional flow features of the porous media and take advantage of simple analytical solutions of flow passing through an equivalent flow model. The Kozeny–Carman equation was realised for the Darcy flow in a homogeneous medium according to an equivalent hydraulic diameter model^35–43^. The hydraulic diameter method can be used for deriving the Forchheimer equation^39^. Ergun’s equation is the most commonly used relationship among various correlations of this type^17,34^. In the preliminary capillary model, Scheidegger^13^, Rumer and Drinker^40^ and Blick^41^ represented the idealised pore geometry as capillaries in series and parallel or as packed beds of spheres. Comiti and Renaud^42^ eventually suggested a cylindrical capillary model according to the pore Reynolds number ($R{e}_{p}$) and the pore friction factor (${f}_{p}$), as demonstrated in Eq. (1), where *v* is the interstitial flow velocity passing through actual pore paths whose average length is defined by *Le*. Notably, Comiti and Renaud^42^ used the pore friction factor (${f}_{p}=\frac{{D}_{p}}{2\rho {v}^{2}}\frac{\Delta P}{{L}_{e}}$), which is a quarter (i.e. 16) of the general friction factor ($f=\frac{2D}{\rho {v}^{2}}\frac{\Delta P}{{L}_{e}}$) defined for cylindrical pipe flows (i.e. 64).

1 $${f}_{p}R{e}_{p}=16+0.1936\cdot R{e}_{p},\quad where\;{f}_{p}=\frac{{D}_{p}}{2\rho {v}^{2}}\frac{\Delta P}{{L}_{e}}, R{e}_{p}=\frac{\rho v{D}_{p}}{\mu }, {D}_{p}=\frac{4\varnothing }{{a}_{vd}\left(1-\varnothing \right)}$$

Theoretical suggestions according to Eq. (1) were compared with several experimental results for packed beds of spheres^42^, cylinders^43^, polyhedrons^44^, plates^45^ and sands^46^. A good agreement between the predicted and experimental data was observed because most of the other correlations were only applicable to a few types of structural configurations of porous media^17,42^. Therefore, the Comiti capillary model according to Eq. (1) can be a promising contender for the flow analyses in both the non-Darcy flow regimes in proppant packs because all the fundamental variables in Eq. (1) can be easily calculated (except tortuosity). However, it should be noted that all the grains used in the previous comparisons were almost single-sized and uniformly shaped. Otherwise, various types of proppants are typically mixed with diverse soil grains of varying sizes and shapes in genuine hydraulic fractures. Furthermore, the proppants and grains can become semi-consolidated owing to the surrounding formation stresses such that the internal flow paths can further be deformed and the resulting geometric and structural changes severely impact the flow performances. Sequin et al.^25^ reported that the packs of synthetic foams (similar to a semi-consolidated pack) indicated various fluctuations in laminarising behaviours (nonlinearity). Therefore, the Comiti model should be evaluated for mono-sized proppant packs as well as for mixed packs with multi-size proppants to confirm its applicability to real proppant packs flows. Furthermore, semi-consolidated proppant packs (similar to foam materials) and various tortuosity (flow direction) cases should be investigated during the examinations.

Section III presents the 182 pore-scale direct numerical simulations conducted to examine the applicability of the Comiti model to simple mono-size and complex multi-size packs. Subsequently, the turbulent friction factor in the logarithmic functional form is proposed in Section IV to obtain a general correlation applicable to the non-Darcy flow analyses, particularly beyond the Forchheimer regime.

## Application of the effective diameters of porous media to the Comiti equation

Before examining the applicability of the Comiti capillary model for several proppant packs, the physical definitions of pore friction factor and pore Reynolds number presented in Eq. (1) are confirmed. Because the Comiti equation was derived on the basis of a cylindrical capillary model, the numeric value of ‘16’ in Eq. (1) must correspond to the friction constant (${f}_{p}R{e}_{p}$) of a normal circular cylinder in the laminar flow regime; notably, the pore friction factor (${f}_{p}=\frac{{D}_{p}}{2\rho {v}^{2}}\frac{\Delta P}{{L}_{e}}$) was simply defined as a quarter of the general friction factor ($f=\frac{2D}{\rho {v}^{2}}\frac{\Delta P}{{L}_{e}}$) in the Comiti equation. Moreover, according to a recent definition by Shin^47^, it was indicated that the laminar friction constant, which was defined on the basis of the hydraulic diameter of porous media (${D}_{h}$, i.e. pore diameter in the Comiti equation), cannot be the same as that of the circular cylinder; $fR{e}_{{D}_{h}}\ne 64$. Therefore, the numeric value presented in Eq. (1) cannot be exactly ‘16’ when the pore friction factor is defined on the basis of the hydraulic diameter.

For reference, we can recall that the hydraulic diameters of non-circular ducts can be easily determined using only the basic geometric relations (quantitatively equivalent). However, to be reasonably (qualitatively equivalent) integrated to conventional viscous pipe flow theories, the hydraulic diameters must be converted to the corresponding effective diameters by adopting each friction constant ratio ($\xi$) of non-circular ducts^48^. Therefore, based on the Kozeny’s hydraulic diameter (${D}_{h}$), tortuosity ($T$) and permeability ($k$) definitions^28^, Shin^47^ presented the effective diameters of porous media (${D}_{e}$), shown in Eq. (2), and suggested replacing the hydraulic diameter with ${D}_{e}$ when a circular cylinder model (${f}_{e} R{e}_{e}=64$) is adopted in porous flow analyses. Notably, the effective friction factor (${f}_{e}$) of porous media was described here using the general Darcy–Weisbach relation derived for cylindrical conduits; thus, ${f}_{e}=4{f}_{p} at {D}_{e}={D}_{p}; \therefore {f}_{e}R{e}_{e}=4{f}_{p}R{e}_{p}=64$^37,47,48^.

2 $${D}_{e}=\frac{{D}_{h}}{{\left(T\cdot \xi \right)}^\frac{1}{2}} \& \overline{{D }_{e}}={D}_{e}\cdot T,\quad where\;{D}_{h}=\frac{4\varnothing }{{S}_{s}}=\frac{4{D}_{g}\varnothing }{{C}_{s}\left(1-\varnothing \right)}={D}_{p}, T=\frac{L}{{L}_{e}}=\frac{u}{\varnothing v},$$

$$\xi =\frac{{f}_{v}R{e}_{v}}{{f}_{e}R{e}_{e}}=\frac{{f}_{v}R{e}_{v}}{64}, {f}_{v}=\frac{2{D}_{h}}{\rho {v}^{2}}\frac{\Delta P}{L}, R{e}_{v}=\frac{\rho v{D}_{h}}{\mu }, {f}_{e}=\frac{2{D}_{e}}{\rho {v}^{2}}\frac{\Delta P}{{L}_{e}} \& R{e}_{e}=\frac{\rho v{D}_{e}}{\mu }$$

Here, the tortuosity ($T$) concept was introduced to distinguish the extremely different flow aspects due to varying and isentropic pore flow paths in each flow direction, i.e. the ratio of the porous medium length ($L$) to the real pore flow path (${L}_{e}$). Moreover, *u* represents the apparent flow velocity of a porous medium and the friction constant ratio ($\xi$) is the ratio of laminar friction constant (${f}_{v} R{e}_{v}$) of the non-circular cross section to that of a circular cylinder^47,48^. Additionally, based on the superficial velocity, (${v}_{s}=\frac{u}{\varnothing }\ne v$) the superficial effective diameter ($\overline{{D }_{e}}$) was defined as another type of effective diameter, using the physical variables measured in the Darcy flow regime with cylindrical friction constant and the same pressure drop along the medium length ($L$)^47^. Consequently, as indicated in Eq. (3), the Comiti equation is modified by replacing the pore diameter in Eq. (1) with the effective diameter of porous media and using the relations presented in Eq. (2). Furthermore, the modified Comiti equation shown in Eq. (3) is converted to the respective correlations of total pressure drop ($\Delta P$), permeability ($k$) and tortuosity ($T$), as demonstrated in Eq. (4). Here all the correlations presented in Eq. (4) were defined on the basis of the superficial diameter ($\overline{{D }_{e}}$), which is easily calculated using the Darcy permeability (${k}_{Darcy}$), because it can be measured only in the Darcy flow regime.

3 $$\therefore {f}_{p}R{e}_{p}=16+0.1936\cdot R{e}_{p},\quad where {f}_{p}\equiv \frac{{D}_{e}\cdot {\varnothing }^{2}{T}^{3}}{2\rho {u}^{2}}\frac{\Delta P}{L} \& R{e}_{p}\equiv \frac{\rho u{D}_{e}}{\mu \cdot \varnothing T}$$

4a $$\therefore \Delta P=\left(16+0.1936\cdot \frac{\rho u\overline{{D }_{e}}}{\mu \cdot \varnothing {T}^{2}}\right)\frac{2\mu uL}{\varnothing \overline{{D }_{e}^{2}}} \because \overline{{D }_{e}}={D}_{e}\cdot T \& {k}_{Darcy}=\frac{\varnothing \cdot {\overline{{D }_{e}}}^{2}}{32}$$

4b $$\therefore k=\frac{\varnothing \overline{{D }_{e}^{2}}}{2\left(16+0.1936\cdot \frac{\rho u\overline{{D }_{e}}}{\mu \cdot \varnothing {T}^{2}}\right)} or {T}^{2}=\frac{0.1936\cdot \frac{\rho u\overline{{D }_{e}}}{\mu \cdot \varnothing {T}^{2}}}{\left(\frac{\varnothing \overline{{D }_{e}^{2}}}{2k}-16\right)} \because u=\frac{k}{\mu }\frac{\Delta P}{L}$$

Therefore, the vital viscous flow variables, such as permeability and pressure variations can be determined regardless of flow regime changes. For reference, the tortuosity correlation presented in Eq. (4b) is interesting because the denominator of the correlation must be zero in the Darcy flow regime ($\because k={k}_{Darcy}=\frac{\varnothing \cdot {\overline{{D }_{e}}}^{2}}{32}$), making this an incompatible equation. However, the permeability ($k$) values estimated using Eq. (4b), after reaching the Forchheimer regime, must be different from the Darcy permeability ($\because {k\ne k}_{Darcy}$) values. Thus, the tortuosity equation can provide meaningful values after reaching the Forchheimer regime when the permeability is measured under the non-Darcy flow conditions and then, a specific tortuosity value, which shows the best match in either pressure difference ($\Delta P$) or permeability ($k$) through trials, can be the estimated tortuosity of target porous medium. Considering that tortuosity is an intricate geometric property that is very difficult and expensive to achieve targeting real porous media, Eq. (4b) could be alternatively used for tortuosity estimations.

Finally, using Eqs. (3) and (4), we can examine the applicability of the Comiti capillary model to various proppant packs. For the examinations, two types of pore-scale simulation (PSS) models whose microscale grid systems, numerical settings and flow conditions were already verified in a previous investigation^31^ were adopted. The original model of ‘mono-size proppant pack’ was considered to exhibit a simple structured shape, such as a set of parallel plates, whose length, width and aperture (height) were initially set to 4 × 4 × 0.5 mm^3^ and filled with five layers of identical spherical beads (see Fig. 7 and Table 1 in the Method section “M1. Summary of the pore‑scale simulations”). Another five porous medium models adopted to mimic the mixed packs with multi-size proppants (‘multi-size proppant packs’); these packs were assumed to have the same set of parallel plates filled with five layers of three differently sized microbeads in staggering arrays (Fig. 8and Table 2 in the Method section M1. Summary of the pore‑scale simulations).

**Table 1:** Summary of geometric variables of the five mono-size pack models.

	(A) Thickest	(B) Thick	(C) Base	(D) Thin	(E) Thinnest
$h \left(\mathrm{mm}\right])$	0.5	0.475	0.45	0.425	0.4
${S}_{S} ({\mathrm{mm}}^{-1})$	35,265.5200	35,126.0474	34,984.7167	34,847.1118	34,712.5563
$\varnothing$	0.45556375	0.430643421	0.406994444	0.384579412	0.363398438
${D}_{h}\left(m\right)$	5.16724E−05	4.90398E–05	4.6534E−05	4.41448E−05	4.18752E−05
Cell counts	113,441,669	87,304,720	75,773,022	67,823,581	62,381,776

Ref: the final cell numbers of the unstructured tetrahedral grid systems of each model were identified using trials of different grid resolutions.

**Table 2:** Summary of geometric variables of the five multi-size pack models.

	(A) Thickest	(B) Thick	(C) Base	(D) Thin	(E) Thinnest
$h \left(\mathrm{mm}\right)$	0.5	0.475	0.45	0.425	0.4
${S}_{S} ({\mathrm{mm}}^{-1})$	41,012.6288	38,188.9000	35,067.1722	31,881.5259	28,902.8064
$\varnothing$	0.21751125	0.192022368	0.169858333	0.150432353	0.133447031
${D}_{h}\left(\mathrm{m}\right)$	2.12141E−05	2.01129E−05	1.93752E−05	1.88739E−05	1.84684E−05
Cell Counts	150,618,936	148,876,948	105,605,578	77,367,108	60,970,350

Ref: grid resolutions (minimum and maximum face-area and cell-volume) were changed to several models owing to convergence issue.

Therefore, 182 experiments were performed with Ansys–Fluent commercial software (Ansys Co. U.S.) to test the applicability of the Comiti model using various PSS models with wide ranges of porosity (13.3%–45.6%) and permeability (0.01–18.07 D) ranges, five mono-size pack models and two series of ten mixed pack models with multi-size proppants in two orthogonal flow directions. The fluid was presumed to be pure liquid water with a standard density and viscosity of 998.2 kg/m^3^ and 0.001003 kg/ms, respectively. The walls and surfaces of the solid bodies were assumed to be completely smooth and isothermal with no-slip conditions. The final cell numbers of each PSS model were identified using trials of different grid resolutions to overcome both convergence and accuracy issues. More detailed information regarding the PSS models and simulations are presented in the Method section “M1. Summary of the pore‑scale simulations”. Moreover, main analysis results are provided in the ‘Supplemental Data’ with an excel file comprising original PSS data. Table S1 (in the ‘Supplemental Material’) summarises the main variables observed under the laminar flow conditions for each PSS case, including pressure drop ($\Delta P$), average interstitial flow velocity ($v$) through pore paths and the calculated effective diameter and tortuosity using the primitive properties. According to flow velocity increment, all the variations in pressure drop, permeability and effective Reynolds number are obtained from each PSS result directly and then listed in Tables S2–4 (in the ‘Supplemental Material’).

**Figure 1:**
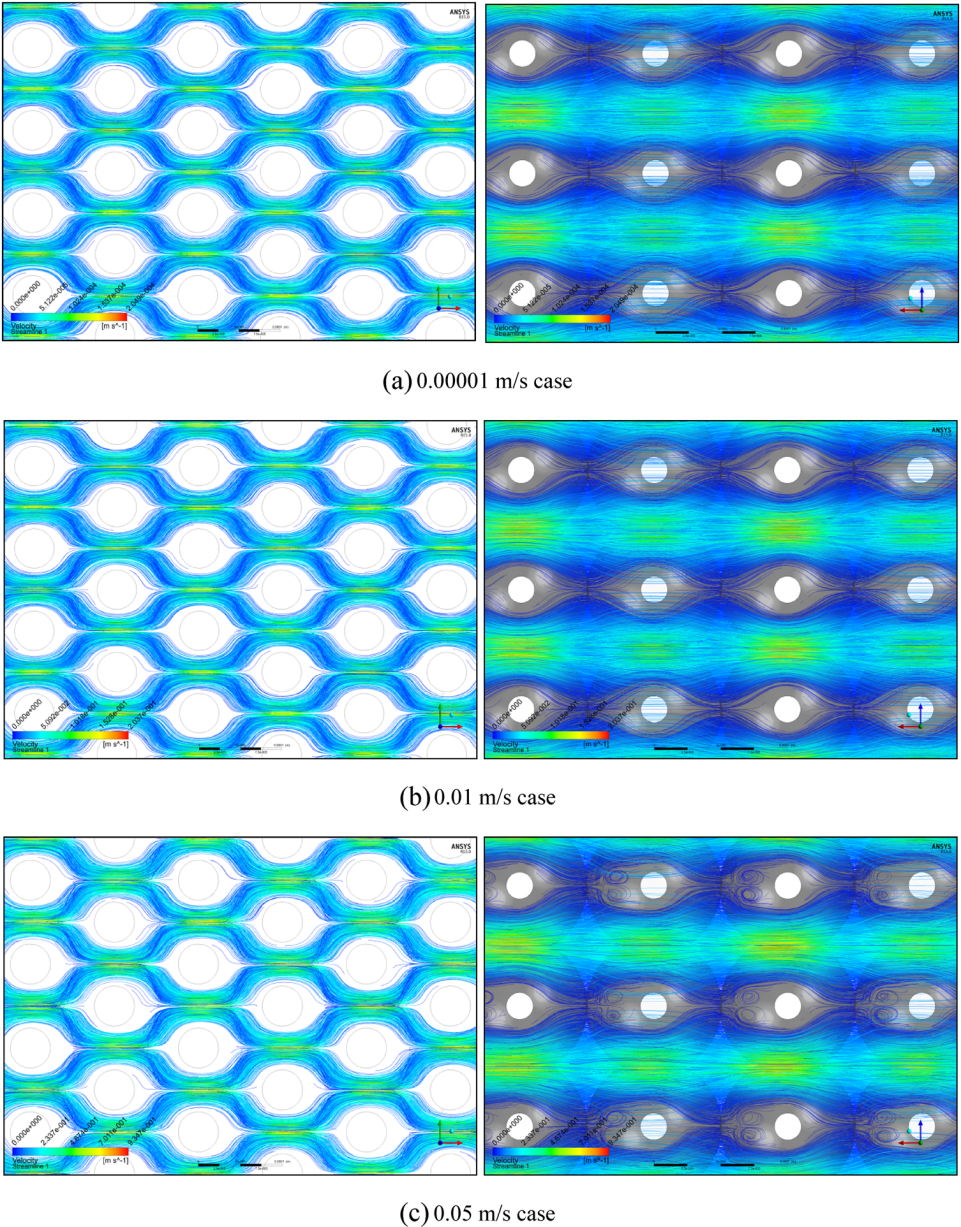

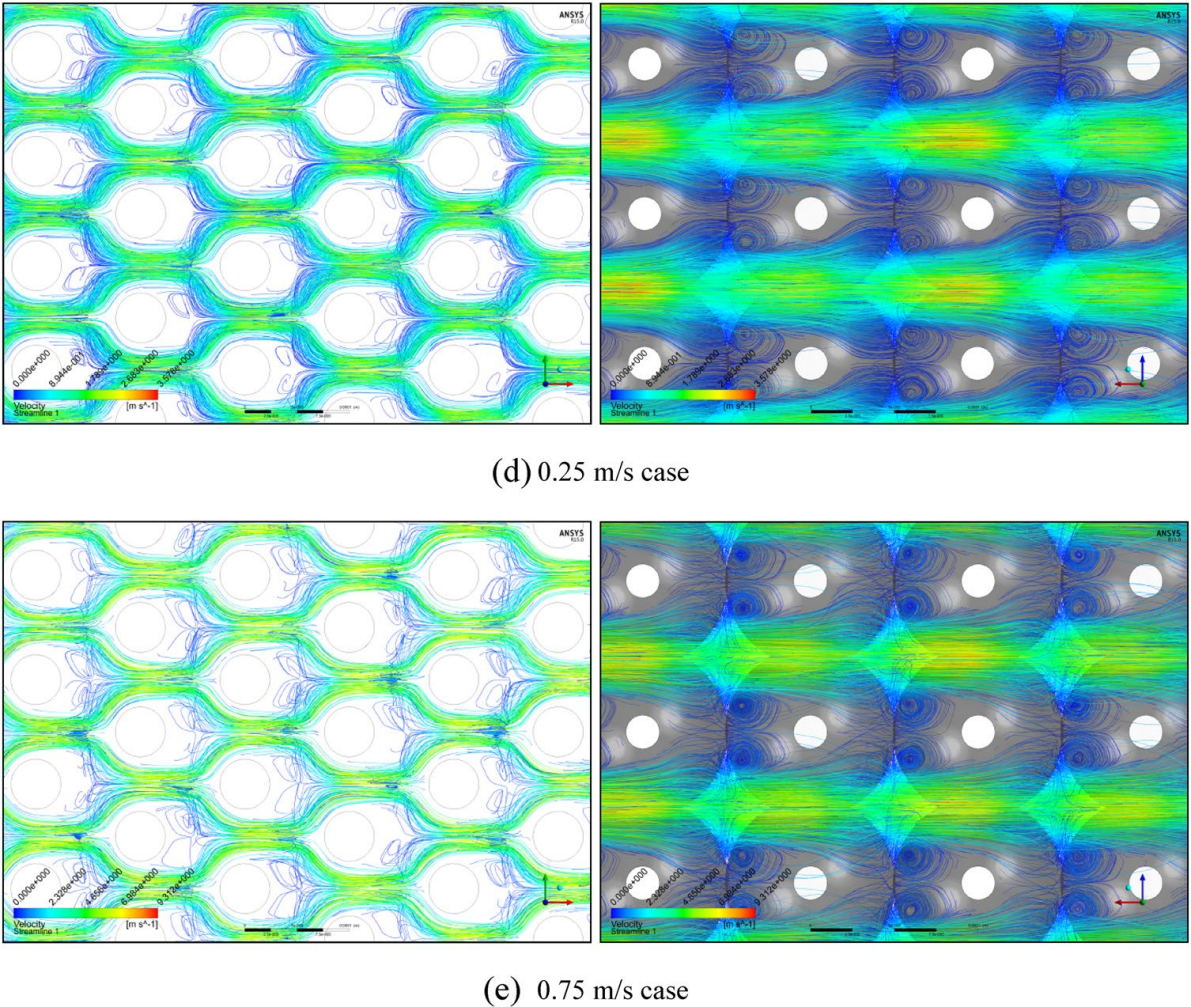
Streamline distributions of the mono-size pack models from **(a)** 0.00001 to **(e)** 0.75 m/s conditions, focusing on the centre parts in the X–Y (left) and X–Z (right) planes.

Subsequently, under five representative velocity conditions, the streamline distributions of the mono-size base model are presented in Fig. 1. Here, the streamlines were generated on the basis of 200,000 uniformly distributed streamline seeds for each PSS case. Figure 1a presents the smooth and continuous flows within the Darcy flow regime, confirmed by permeability rigidity; these flows seem to be correspond to the (1) Darcy (creeping flow) regime defined by Dybbs and Edwards^26^. Compared with Fig. 1a, Fig. 1b shows only minimal variations, such as slight reductions in the streamlines and locally observed few streamline cuts, i.e. (2) weak inertial flow regime^26^. More instability, including globally identified flow cuts and few eddy structures, can be observed in Fig. 1c; i.e. (3) unsteady laminar (Forchheimer) regime^26^. Finally, strong instability, such as many streamline cuts and three-dimensional vortices, is steeply strengthened, as shown in Fig. 1d,e, placing it in the turbulent regime; i.e. (4) highly unsteady and chaotic flow regime^26^. In summary, the observations by Dybbs and Edwards^26^ were rechecked using Fig. 1 and all the regimes from (1) Darcy to (3) Forchheimer can be classified according to various types of the laminar flows while turbulent behaviours are widely observed in (4) chaotic flow regime.

**Figure 2:**
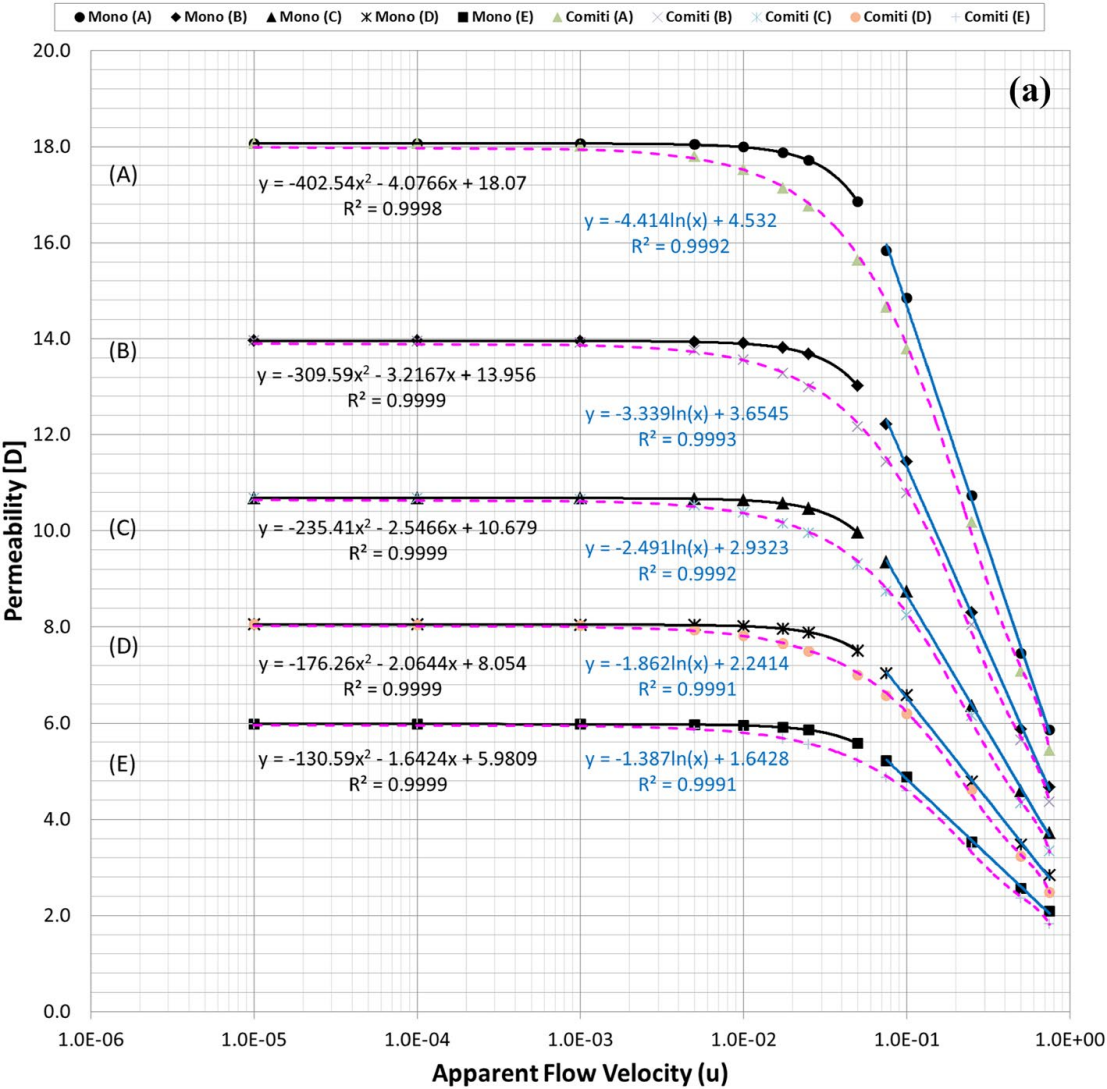

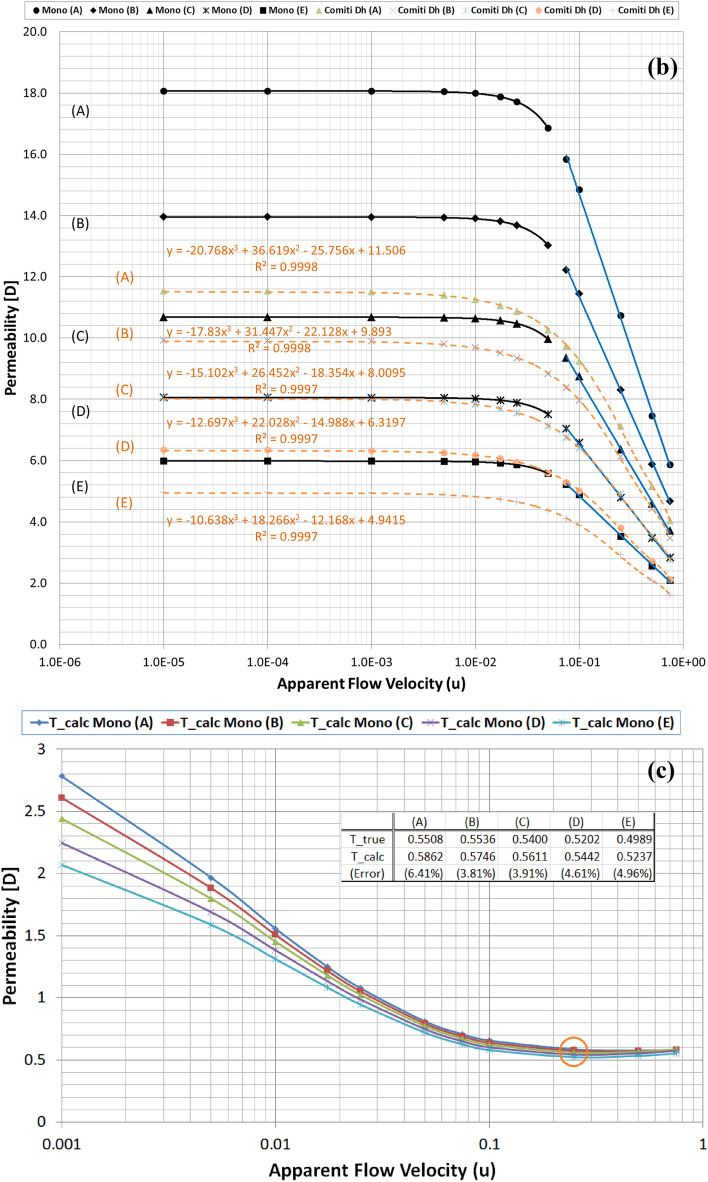
**(a)** Comparisons of the permeability variations of the mono-size pack models using the modified Comiti equation (dotted pink lines) (Eq. 4b) and those directly obtained from each PSS analysis (solid black and blue lines) relative to the apparent flow velocity increments. **(b).** Comparisons of the permeability variations of the mono-size pack models using the original Comiti equation (dotted orange lines) based on the pore (hydraulic) diameter presented in Eq. (1) and the data directly obtained from each PSS analysis (solid black and blue lines). **(c).** Tortuosity variations of the mono-size pack models, estimated using the tortuosity equation (Eq. 4b).

Figure 2a plots the calculated permeability variations of each mono-sized pack model using the modified Comiti equation in Eq. (4b) over the original PSS results shown in semi-log plots. Hence, it is verified that the modified Comiti equation results in reasonable permeability variations for the packed bed comprising mono-size proppants from the Darcy to non-Darcy flow regimes. Moreover, the equation indicates satisfactory outcomes not only in the original proppant pack ((A) thickest model) but also in the semi-consolidated packs ((B)–(E) models). However, the original Comiti model shown in Eq. (1) defined using the pore diameter generates huge differences (Fig. 2b). Furthermore, each best regression curve (showing the highest R-squared (*R*^2^) values, i.e. determinant coefficients) is displayed in Fig. 2a to distinguish the different aspects between the laminar (Darcy to Forchheimer) and turbulent (beyond the Forchheimer) regimes. The black lines in Fig. 2a represent the regression results in the laminar regimes (Fig. 1a–c) whereas the blue lines are those in the turbulent regime (Fig. 1d,e). As reported in former studies, we confirm here that the permeability varied in quadratic until the Forchheimer regime^17,20,26^ then changed to logarithmic beyond the Forchheimer regime^11,13,26^.

Figure 2c shows the estimated tortuosity variations using the tortuosity equation presented in Eq. 4b. The best matches in permeability (judged by checking the calculation errors after the Forchheimer regime) were observed at a velocity of 0.25 m/s while showing the inflexion aspects of the estimated tortuosity. (see in the red circle marked in Fig. 2c). The calculated values (${T}_{calc}$) are illustrated in the figure with errors (3.8–6.4%) with respect to the true values (${T}_{true}$) directly determined from each PSS case. Overall, the tortuosity equation presented in Eq. 4b produces satisfactory outcomes for all the mono-size pack models. This is a remarkable result, given that the actual tortuosity estimations are only applicable to the materials with simple pore structures in small size^24,25^ requiring complex and expensive procedures, whereas this method based on Eq. 4b can be applied to large media including actual formations, by measuring pressure drops in the non-Darcy flow regimes.

**Figure 3:**
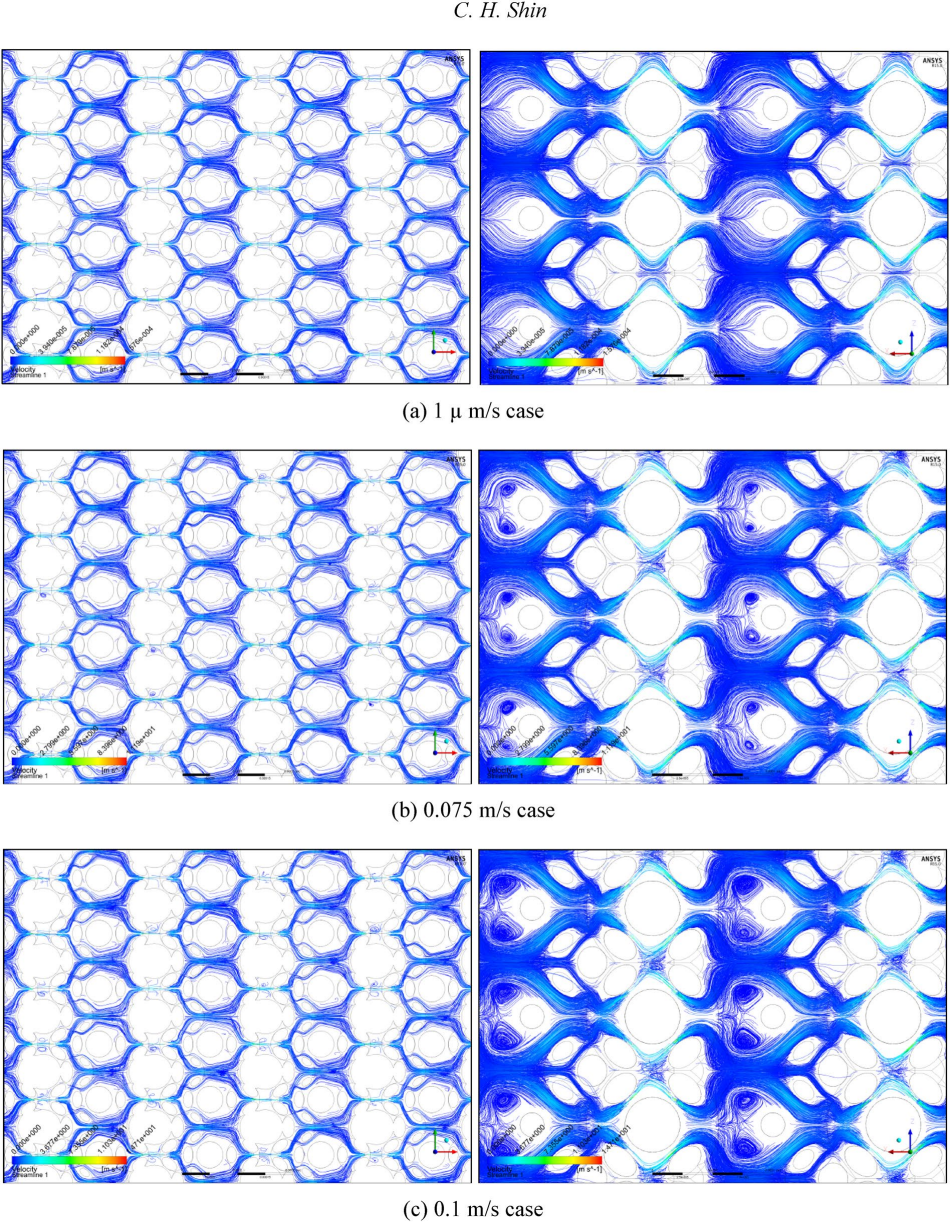
Streamline comparison of (C) multi-size base model in the three X-directional flow velocity conditions focusing on the centre parts in the X–Y (left) and X–Z (right) planes.

**Figure 4:**
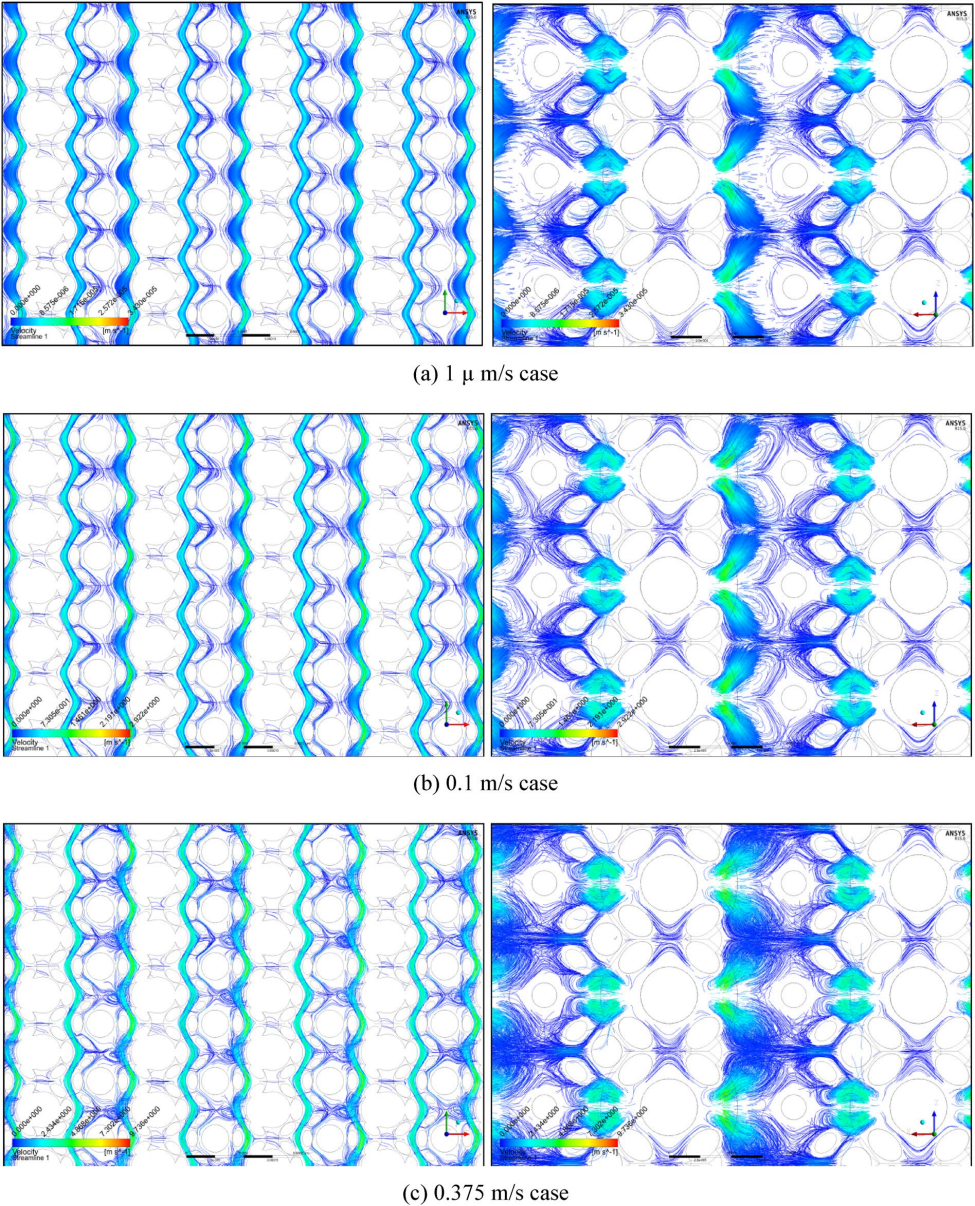
Streamline comparison of (C) multi-size base model in the three Y-directional flow conditions focusing on the centre parts in the X–Y (left) and X–Z (right) planes.

**Figure 5:**
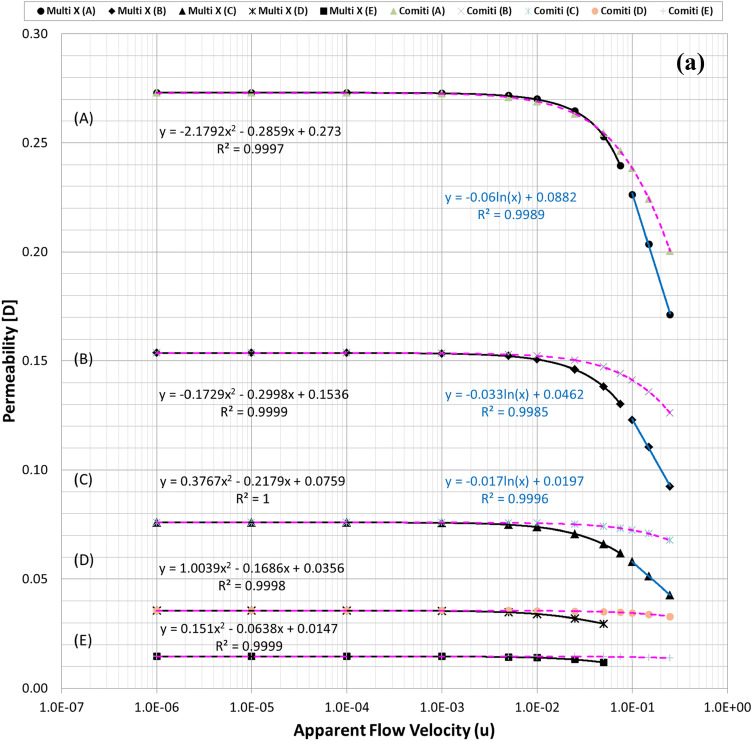

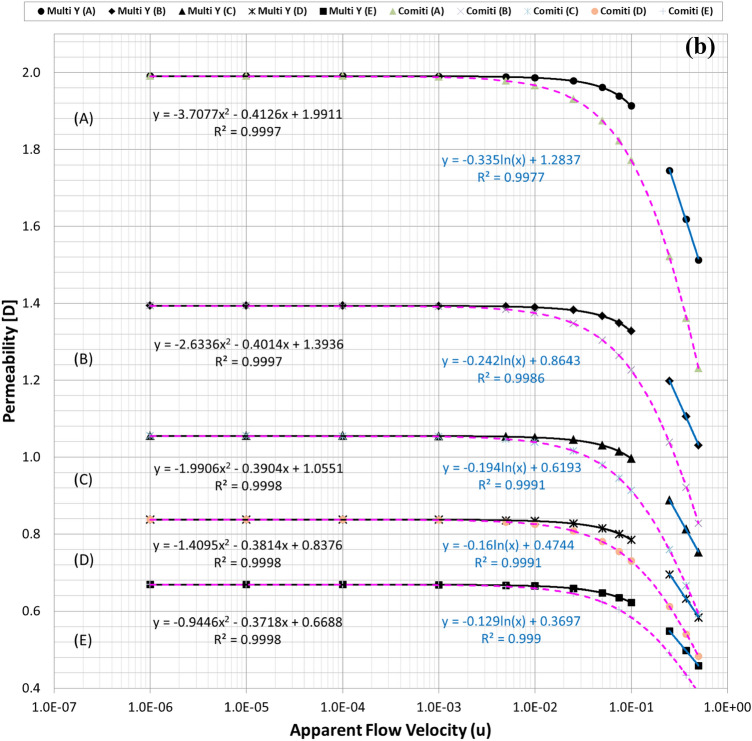
**(a)** Comparisons of the permeability variations of the multi-size pack models in the X-directional flow conditions using Eq. (4b) (dotted pink lines) and those directly obtained from each PSS analysis (solid black and blue lines). **(b)** Comparisons of the permeability variations of the multi-size pack models in the Y-directional flow conditions using Eq. (4b) (dotted pink lines) and those directly obtained from each PSS analysis (solid black and blue lines).

Figures 3 and 4 summarise the individual streamline distributions in each PSS case for multi-size pack models in the X- and Y-directional conditions, respectively. Figure 5a,b plot the permeability variations of each multi-size pack estimated using the modified Comiti equation in semi-log plots. The mixed packs illustrated in Figs. 3 and 4 present similar aspects as those of the mono-size packs shown in Fig. 1 in terms of flow regime changes. However, very complex flow patterns and narrowed pores owing to the multi-scale, staggered and embedded grains were observed in both directional flow cases. Subsequently, it can be observed from Fig. 5a,b that both results of the permeability variations estimated using the modified Comiti equation are quite poor. Furthermore, the errors are increased in more consolidated (thinner) media and higher flow velocity conditions, both within and beyond the Forchheimer regime. Therefore, the Comiti capillary model gives reasonable results regarding the mono-size packs, such as the simply deformed media; however, the modified Comiti equation does not apply to the complex and mixed packs with multi-size proppants. This implies that the first numeric value ‘16’ in the Comiti equation can be fixed by adopting the effective diameter of porous media; however, the other numeric value ‘0.1936’ is not constant. In addition, the tortuosity equation presented in Eq. 4b converted from the modified Comiti equation cannot be introduced to the mixed proppant packs owing to the poor correlations.

## Introduction of the turbulent friction factor for flow analysis beyond the Forchheimer regime

This study initially aimed to verify whether the Comiti capillary model can be generally applied to both the non-Darcy flow regimes, including the Forchheimer regime and the turbulent regime. Consequently, the modified Comiti equation was confirmed to be valid for applications to the mono-size simple proppant packs but invalid for the mixed complex packs comprising multi-size proppants, specifically showing very large errors beyond the Forchheimer regime. Moreover, we can recall that criteria generally identifying each flow regime change are yet to be presented. Therefore, a new reliable correlation should be presented, which can be used for various types of real proppant packs regardless of flow regime changes (i.e. not requiring any criteria for flow regime classification, particularly beyond the Forchheimer regime).

Figures 2a, 5a,b demonstrate that permeability fluctuates in quadratic until the Forchheimer regime but then changed to logarithmic beyond the Forchheimer regime. Moreover, the logarithmic variation of the turbulent friction factor in the turbulent flow regime is fundamental in conventional viscous flow analyses of normal conduits^48–51^. Therefore, the logarithmic function definition of the turbulent friction factor could be essential (more reasonable) for the porous flow analyses beyond the Forchheimer regime. Accordingly, this study attempts to define the logarithmic turbulent friction factor (${f}_{turb}$) of porous media by adopting the effective variables shown in Eq. (2) and then examines whether the new non-Darcy correlation based on the turbulent friction factor can be used more generally for the non-Darcy flow analyses compared with the Ergun-type quadratic equations including the Comiti model.

We first introduce the basic concept shown in Eq. (5), which was initially proposed for deriving Ergun’s equation^21,27^, by assuming an equivalent cylindrical flow model defined using the superficial diameter ($\overline{{D }_{e}}$) and the medium length ($L$). Here, the total shear stress (${\tau }_{total}$) created by both the inertial and viscous flow effects on a packed bed must be equivalent to the sum of the laminar (${\tau }_{lam}$) and turbulent (${\tau }_{turb}$) shear stresses. Consequently, the total pressure drop ($\Delta {P}_{total}$) can be defined as a function of the laminar and turbulent friction factors, as presented in Eq. (6). Notably, the laminar pressure drop ($\Delta {P}_{lam}$) is defined as the pressure drop resulted from only the laminar flow effects (excluding the turbulent effects even in the turbulent regime, on purpose), by adopting the Darcy permeability (${k}_{Darcy}$), which is defined only in the Darcy flow regime.

5 $$\frac{{F}_{drag}}{S}={\tau }_{total}={\tau }_{lam}+{\tau }_{turb}\quad where\;{F}_{drag}=\Delta {P}_{total}\cdot \frac{\pi {\overline{{D }_{e}}}^{2}}{4} \& S=\pi \overline{{D }_{e}}L$$

$$\therefore \Delta {P}_{total}=\frac{4L}{\overline{{D }_{e}}}\left({\tau }_{lam}+{\tau }_{turb}\right) \quad where\;{\tau }_{lam}=\rho {v}^{2}\cdot \frac{{f}_{lam}}{8} \& {\tau }_{turb}=\rho {v}^{2}\cdot \frac{{f}_{turb}}{8}$$

6 $$\therefore \Delta {P}_{total}=\Delta {P}_{lam}\left(1+\frac{{f}_{turb}}{{f}_{lam}}\right) \because {\tau }_{lam}=\frac{\overline{{D }_{e}}\cdot \Delta {P}_{lam}}{4L}$$

$$where {f}_{lam}=\frac{2\overline{{D }_{e}}\cdot \Delta {P}_{lam}}{\rho {v}^{2}\cdot L}=\frac{2\overline{{D }_{e}}\cdot \Delta {P}_{lam}}{\rho {u}^{2}\cdot L}\cdot {\varnothing }^{2}{T}^{2}, \Delta {P}_{lam}=\frac{32 \mu uL}{\varnothing \cdot {\overline{{D }_{e}}}^{2}} \& {\overline{{D }_{e}}}^{2}=\frac{32\cdot {k}_{Darcy}}{\varnothing }$$

In Eq. (6), for the laminar flow regime, the laminar shear stress (${\tau }_{lam}$) was converted from ${\tau }_{lam}$ shown in Eq. (5) by introducing the laminar effective friction factor (${f}_{lam}$), which is another expression of the effective friction factor (${f}_{e}$) defined in Eq. (2) according to the laminar pressure drop ($\Delta {P}_{lam}$), thus reflecting the laminar friction effect induced from only the laminar flow even in the turbulent regime. The superficial diameter ($\overline{{D }_{e}}$) can be easily determined simply using the Darcy permeability (${k}_{Darcy}$) presented in Eq. (4b).

Conversely, for the turbulent flow regime, the traditional methods such as Moody’s chart^49^ or Colebrook’s equation^50^, which has been extensively used to estimate the turbulent friction factor of normal conduits, can be introduced if we can truly define the equivalent cylinder models. In this study, the Haaland formula^51^, proposed later and widely used as an alternative explicit formula, was chosen to evaluate the turbulent friction factor of porous flow, as demonstrated in Eq. (7). Here, for the porous flow analyses, the effective Reynolds number ($R{e}_{e}$) was adopted mainly on the basis of the effective diameters and hydraulic tortuosity and then redefined as a function of the apparent flow velocity ($u$) because the interstitial flow velocity ($v$) in the turbulent regime cannot be directly determined.

7 $${\left(\frac{1}{{f}_{turb}}\right)}^{0.5}\approx -1.8 \mathit{log}\left(\frac{{C}_{1}}{R{e}_{e}}+{C}_{2}\right) \quad where\;{C}_{1}=6.9, {C}_{2}={\left(\frac{\epsilon /D}{3.7}\right)}^{1.11}$$

$$and\;R{e}_{e}=\frac{\rho v{D}_{e}}{\mu }=\frac{\rho u\overline{{D }_{e}}}{\mu \varnothing } \because T=\frac{u}{\varnothing v} \& \overline{{D }_{e}}={D}_{e}\cdot T$$

In Eq. (7), the terms remaining to be defined for porous media are the two constant terms. If we can deduce a general expression for the constant terms of porous media, then this approach for introducing the logarithmic turbulent friction factor into the non-Darcy flow analyses can be a promising alternative that may be expanded to various types of actual porous media. In the original Haaland formula, the numeric constant (${C}_{1}$) was set to 6.9 and the wall roughness factor term was defined as ${C}_{2}={\left(\frac{\epsilon /D}{3.7}\right)}^{1.11}$. However, the original values and expressions cannot be used for porous media, even the roughness ($\epsilon$) is impossible to define in porous media and the turbulence is the most difficult phenomenon in fluid physics. Hence, an assumption of either reasonable expressions or values for porous media is essential. Therefore, the physical concepts of the two constant terms in the original Haaland formula were reviewed to obtain clues for the remaining constant terms. First, the roughness term was defined according to the characteristic ratio between the wall roughness length and pipe diameter. This could be interpreted as the ratio of the characteristic length of the pore path to the hydraulic diameter of porous media. One of the main representative geometric variables to be considered as the characteristic pore length is the superficial effective diameter because it is the only variable that is solely correlated to the pore flow performance (permeability) with constant values; i.e. ${C}_{2}\approx f(\frac{\overline{{D }_{e}}}{{D}_{h}})$. Therefore, the roughness term can be first assumed as ${C}_{2}=f\left({\left(\frac{T}{\xi }\right)}^{0.5}, \varnothing \right) \because \frac{\overline{{D }_{e}}}{{D}_{h}}=\frac{{D}_{e}T}{{D}_{h}}=\varnothing {\left(\frac{T}{\xi }\right)}^{0.5}$ in porous media.

Second, the value of the numeric constant (${C}_{1}$) must be consistent as 6.9 when the new equation is applied to a circular capillary because ${C}_{1}$ was initially set targeting the general cylindrical conduits (${C}_{1}=6.9$). Finally, the two constants are united into a single correction constant (${C}_{c}$), assuming the above two clues act identically to both the constant terms. This assumption is made because we verified it in the previous section that the last numeric constant ‘0.1936’ in the Comiti quadratic correlation cannot be a universal constant. Moreover, turbulent friction is influenced by the individually variable flow paths (tortuosity and then the superficial diameter) even within an identical porous medium so that either of the constant terms cannot be fixed to a constant in porous media. Consequently, the correction constant is initially considered as a function of porosity, tortuosity and friction ratio (${C}_{c}=\left({\left(\frac{T}{\xi }\right)}^{0.5}, \varnothing \right)$) while restricted by ${C}_{c}=6.9$ when applied to cylindrical capillaries ($\varnothing =T=\xi =1$). The examinations targeting all the three types of 15 PSS models with several combinations of the independent variables of correction constant including the initially assumed expression (${C}_{c}=6.9\cdot \varnothing {\left(\frac{T}{\xi }\right)}^{0.5}$) were conducted to investigate whether a general expression of the correction constant exists.

**Figure 6:**
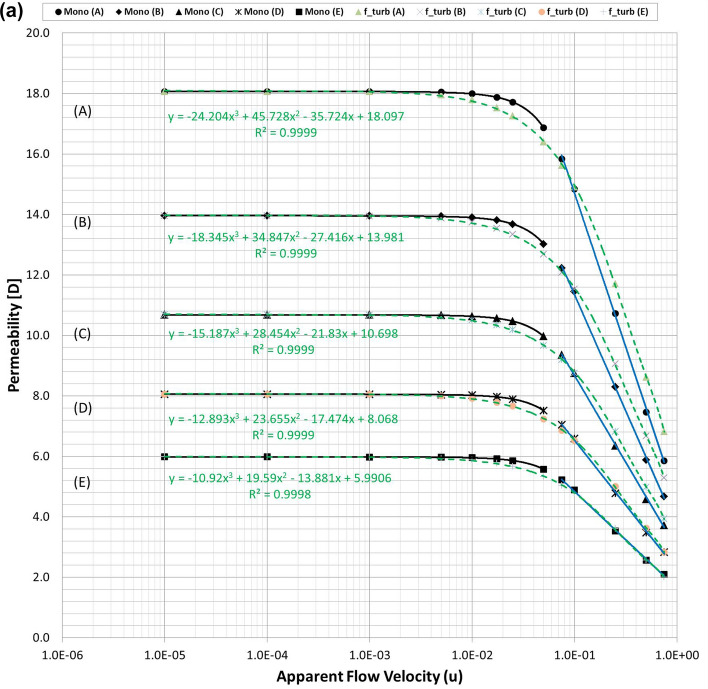

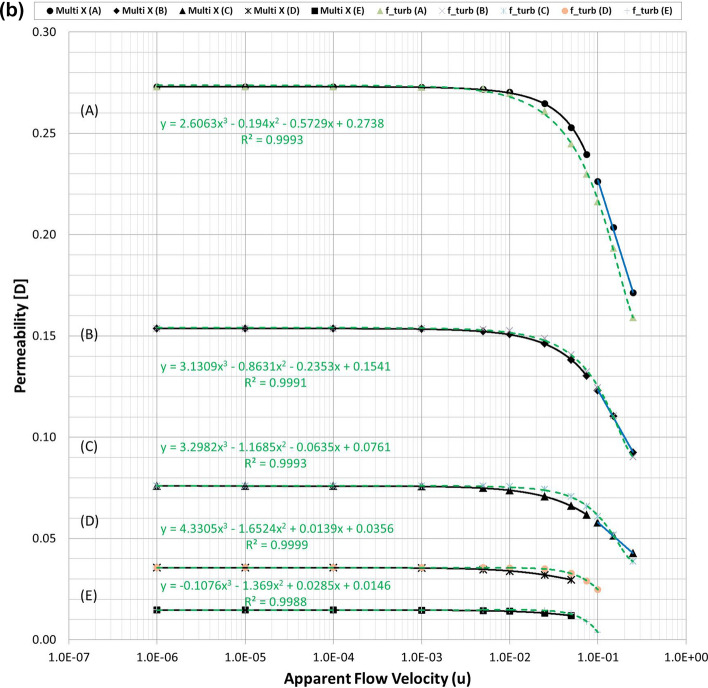

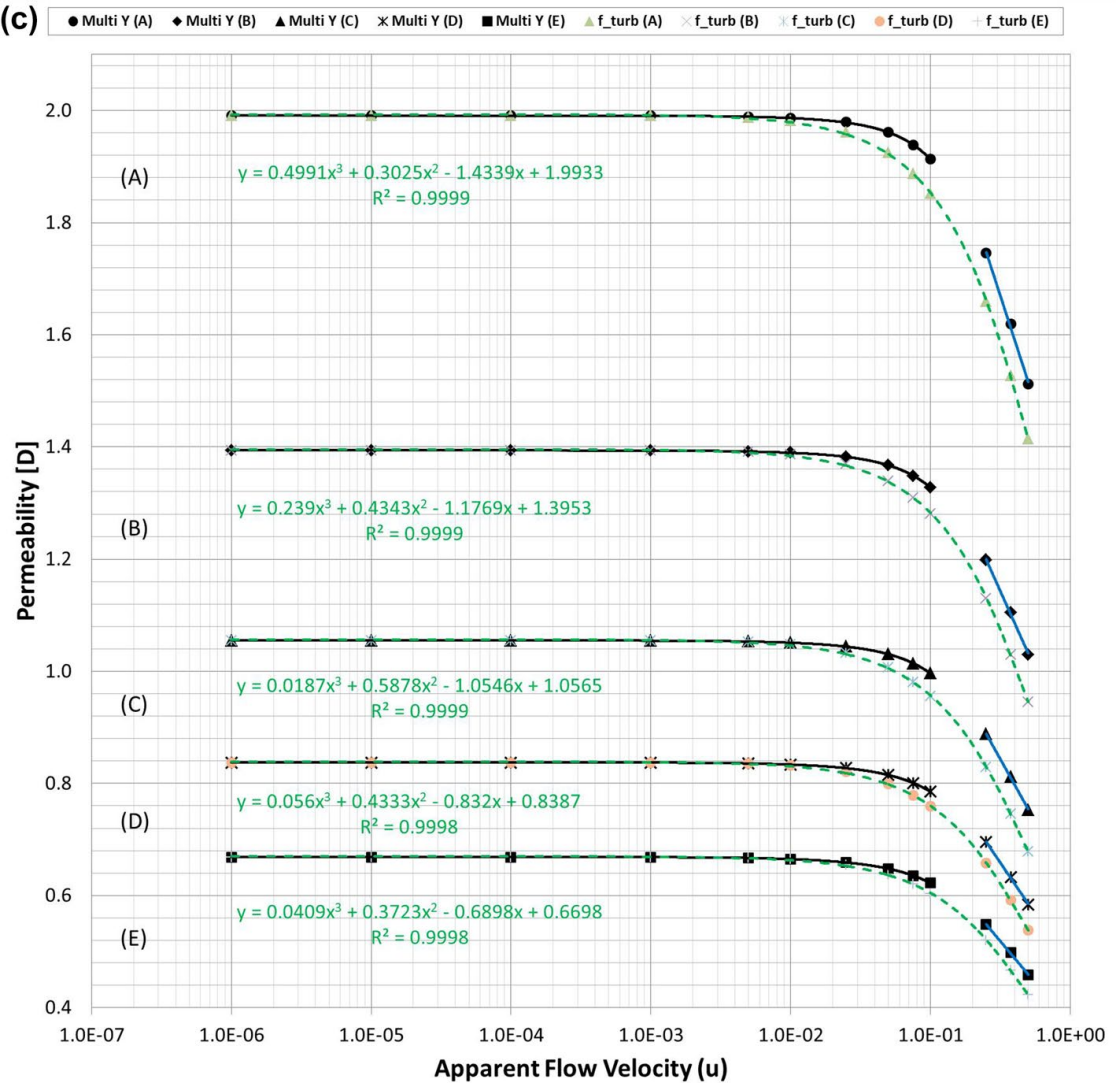
**(a)** Permeability variations of the mono-size pack models were estimated using Eq. (6) (dotted pink lines) and compared with those directly obtained from each PSS analysis (solid black and blue lines) and the regression curves of the estimated permeability for each PSS case. **(b)** Permeability variations of the multi-size packs in the X-directional flow conditions were estimated using Eq. (6) (dotted pink lines) and regression curves for each PSS case. **(c)** Permeability variations of the multi-size packs in the Y-directional flow conditions were estimated using Eq. (6) (dotted pink lines) and regression curves for each PSS case.

Finally, the expression of ${C}_{c}=6.9{\left(\frac{{T}^{\varnothing }}{\xi }\right)}^{0.5}$ presented in Eq. (8) was obtained from data analysis by checking which combination integrated consistently with Eq. (6) to provide reasonable results for the various porous media with wide ranges of porosity, tortuosity and permeability. For three types of PSS models, the resulting total pressure drops calculated using Eqs. (6) and (8) are listed in Table S5 and the permeability variations are plotted in Fig. 6a–c. Figure 6 shows that when Eq. (6) is coupled with Eq. (8), accurate results are obtained in all PSS analysis cases from the Darcy to non-Darcy flow regimes. In addition, the resulting permeability of every PSS case shows the best fits (regressions) in the cubic equation; this is the same as when Forchheimer introduced a cubic law to fit the experimental data^52^. Therefore, we can conclude that the new non-Darcy flow equation, which incorporates the logarithmic, turbulent friction factor and effective variables of porous media, can be more extensively applied to various proppant packs than the Ergun-type quadratic equations including the Comiti model.

8 $${f}_{turb}\approx {\left[1.8\cdot \mathrm{log}\left(\frac{{C}_{c}}{R{e}_{e}}+{C}_{c}\right)\right]}^{-2}\quad where\;{C}_{c}=6.9{\left(\frac{{T}^{\varnothing }}{\xi }\right)}^{0.5} \& {\xi }_{u}=\frac{{f}_{u}R{e}_{u}}{64}=\frac{\xi }{\varnothing T}$$

In summary, a new type of non-Darcy flow equation was successfully presented according to the logarithmic turbulent friction factor based on the effective variables of porous media and the physical properties that can be determined in the Darcy flow regime. Subsequently, the new equation produces accurate results in all the PSS cases while overcoming the critical limitations of the current non-Darcy flow equations, without requiring the determination of the Forchheimer factor and universal criteria for flow changes from the Darcy to beyond Forchheimer regimes. Therefore, the new equation is expected to reasonably apply to various proppant packs regardless of flow regime changes, which is particularly crucial in unconventional oil and gas reservoirs.

## Discussion and conclusions

This study initially aimed to verify whether the Comiti capillary model can be generally applied to both the non-Darcy flow regimes, including the Forchheimer regime and turbulent regime. The modified Comiti equation, which incorporates the effective diameters of porous media, was confirmed to be valid for the mono-size simple proppant packs but invalid for the mixed complex packs comprising multi-size proppants, specifically those showing very large errors beyond the Forchheimer regime.

The turbulent friction factor in the logarithmic function form was then proposed to obtain a general correlation applicable to turbulent flow from the Darcy via Forchheimer. Finally, the equation defined using both the laminar and turbulent friction factors was confirmed to provide accurate results for all the types of proppant pack models with broad ranges of porosity (13.3%–45.6%) and permeability (0.01–18.07 D). In brief, a novel type of the non-Darcy flow correlation is proposed herein that can be widely applied to a variety of porous flow analyses, such as hydraulically fractured wells, particularly beyond the Forchheimer regime that is specifically relevant for flow in proppant packs at practical flow rates.

Considering that the current non-Darcy flow correlations, including the Ergun and Comiti equations, are only applicable to a few similar or simple porous media, the new correlation is expected to be practically used in various proppant packs with broad ranges of geometry and flow properties and a new alternative to be improved not requiring the difficult determinations of Forchheimer factor and universal criteria for flow regime changes beyond Forchheimer regime.

## Method

### M1. Summary of the pore-scale simulations

**Figure 7:**
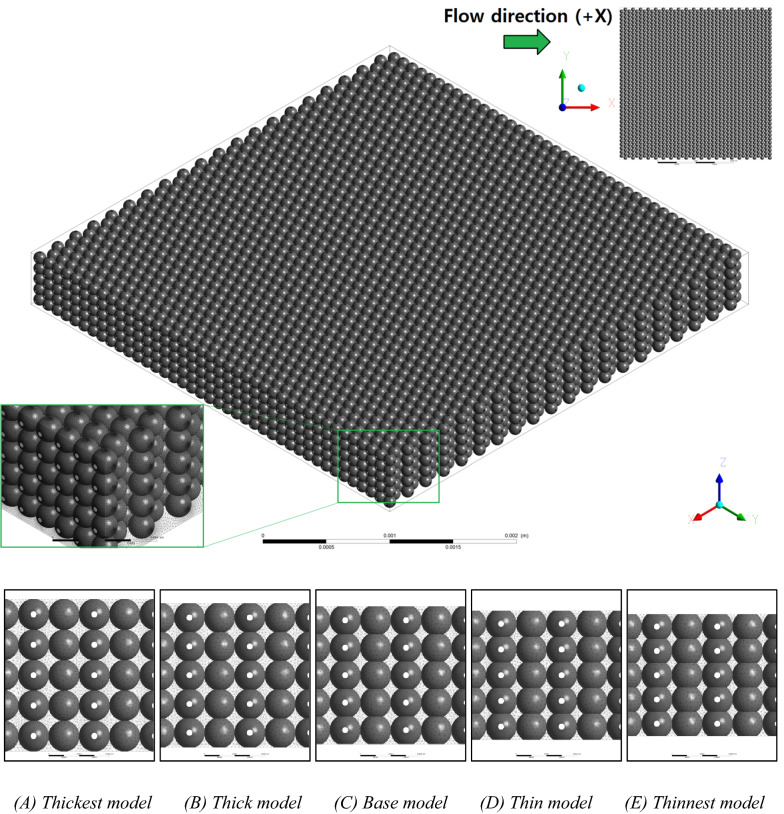
Geometry of the **(A)** thickest model of the mono-size pack model (upper figure) and geometry comparisons in the aperture reductions of five models **(A–E)** in the X–Z plane; total of 7900 microbeads with 0.102 mm uniform diameter (the original diameter of the spheres, excluding the cuts by plates and overlapping with neighbouring beads).

The original model of ‘mono-size proppant pack’ was considered to exhibit a simple structured shape; such as a set of parallel plates, whose length, width and aperture (height) were initially set to 4 × 4 × 0.5 mm^3^ and filled with five layers of the identical spherical beads, as illustrated in Fig. 7. To mimic aperture reductions (semi-consolidation) from the thickest (A) to thinnest models (E), they were reduced into thin models assuming that the beads in each medium were simply and uniformly embedded into adjacent beads and walls. The original (thickest) pack model is the most similar model to commonly packed beds of spheres used in various prior investigations; whereas, the other four reduced models can be regarded as a series of the semi-consolidated proppant packs deformed owing to increased formation stresses (or foam materials). The key geometric features and grid information of the mono-sized pack models are summarised in Table 1. According to the hydraulic diameter and average cell size of the thickest PSS model, the Knudsen number ($Kn$) was checked as ranging from 0.000005 to 0.00025, respectively. The traditional method of computational fluid dynamics applies to the PSS models; $\because Kn\ll 0.1$ were examined^53,54^.

**Figure 8:**
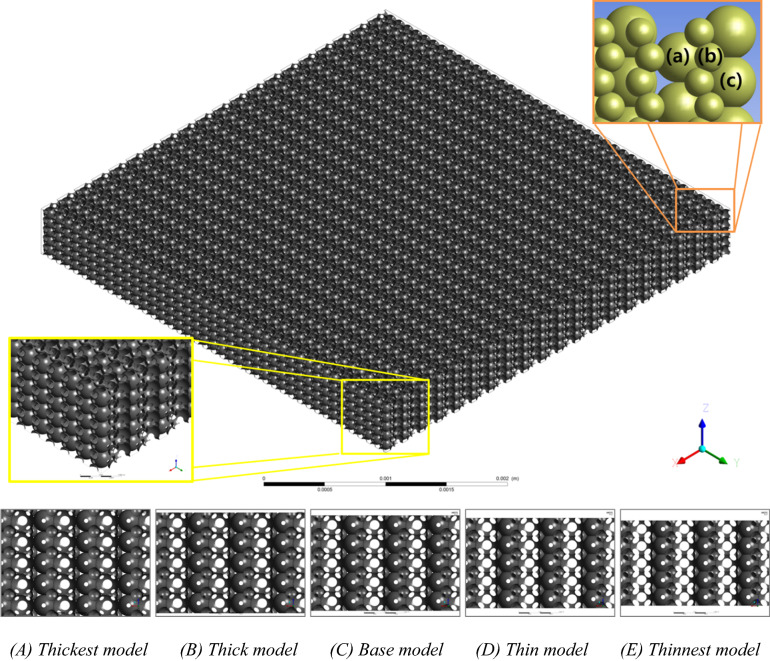
Original geometry of the multi-size pack model (upper figure, (C) base model) after five layers of three differently sized microbeads (a total of 28,160 microbeads) are excluded, along with the five aperture models in the X–Z plane (lower figure, shown in **A–E**). In the left-middle image, the yellow-boxed corner of the model is magnified to provide a more detailed display of medium structures. The upper-right image in the upper figure displays three bead sizes in staggered arrays (radii 51, 30 and 55 µm for **(a)**, **(b)** and **(c)**, respectively).

Another five porous medium models adopted to mimic the mixed packs with multi-size proppants (‘multi-size proppant packs’) were assumed to have the same set of parallel plates filled with five layers of three different sized microbeads in staggering arrays (Fig. 8 and Table 2). Subsequently, to check the tortuosity influences, as shown in Fig. 8 two different flow directions were imposed to the five multi-size pack models. Therefore, to examine the applicability of the modified Comiti equation a total of 15 PSS models in a wide range of porosity (13.3%–45.6%)—5 mono-size pack models and two series of 10 multi-size pack models in two orthogonal flow directions—were initially built. The fluid was presumed to be pure liquid water with a standard density and viscosity of 998.2 kg/m^3^ and 0.001003 kg/ms, respectively. The walls and surfaces of the solid bodies were assumed to be completely smooth and isothermal with no-slip conditions. The second-order upwind scheme and SIMPLE approach were applied for spatial discretisation and pressure–velocity coupling, respectively. The final cell numbers of each PSS model were identified using trials of different grid resolutions to overcome both convergence and accuracy issues.

To investigate the flow variations from the Darcy to non-Darcy (turbulent flow) regime in every PSS model for ‘mono-sized proppant pack’, the 13 apparent flow velocity ($u$) conditions (from 10 µm/s to 0.75 m/s) were set. The apparent velocities were set perpendicular to the inlet section (in the Y–Z plane) and aligned with the X-direction (Fig. 7). Consequently, 65 PSS analyses were performed with Ansys–Fluent commercial software (Ansys Co. U.S.). To consider the flow direction (tortuosity) alterations within an identical medium, the flow direction in the PSS models for ‘multi-size proppant pack’ was set in two directions (X and Y). The apparent velocities were set perpendicular to each inlet section (in the Y–Z and X–Z planes) and aligned with each flow (X and Y) direction (Fig. 8). Thirteen inlet flow velocity conditions from 1 µm/s to 0.5 m/s were applied to the Y-directional flow cases (65 PSS cases). Meanwhile, 12 inlet velocity conditions from 1 µm/s to 0.25 m/s were initially set to the X-directional flow cases, but only 8 velocity conditions from 1 µm/s to 0.05 m/s were applied to the thin and thinnest models because of the rapidly increasing convergence problems owing to the complex and narrow pore structures (52 PSS cases). The convergence criteria were originally set to residuals of less than the order of ${10}^{-8}$ in all momentum equations and the continuity equation. However, the limits were lessened in the order of ${10}^{-7}$ for the ‘multi-size proppant pack’ models.

## Supplementary Information

Supplementary Tables.

## Data Availability

An excel file containing most primitive data, analyses and plots in this study are provided in the ‘Supplemental Data’. The other data that support the findings of this study are available from the corresponding author upon reasonable request.

## Nomenclature

${a}_{vd}$: Dynamic specific surface area: ${a}_{vd}=\frac{{C}_{s}}{{D}_{g}}$
$$C$$: Constant
${C}_{K}$: The Kozeny constant
${C}_{s}$: Kozeny’s shape constant
${C}_{c}$: Correction constant of Haaland equation
$$D$$: Diameter
$${D}_{g}$$: Grain *D*
$${D}_{h}$$: Hydraulic $$D$$
$${D}_{e}$$: Effective $$D$$
$$\overline{{D }_{e}}$$: Superficial $${D}_{e}$$
$${F}_{drag}$$: Drag force
$F, f$: Friction factor (${f}_{v}: f$ defined based on $v$)
$h$: Height of porous medium (model)
$$k$$: Permeability; $$k=\frac{2{D}_{h}^{2}}{{f}_{u} R{e}_{u}} \mathrm{at} u=-\frac{k}{\mu }\frac{\Delta P}{L}$$
$${k}_{C}$$: Carman’s $$k$$
$${k}_{K}$$: Kozeny’s $$k$$
$$L$$: Medium length
${L}_{e}$: Actual flow length
$$P$$: Pressure
$$\Delta P$$: Pressure difference
$$Re$$: Reynolds number
$Re_v$: *Re* based on *v*
$Re_{part}$: *Re* based on particle diameter
*S*: Surface area
*S*_*S*_: Specific surface area
*T*: Hydraulic tortuosity ($$T=L/{L}_{e}$$)
*u*: Apparent flow velocity through a medium
*v*: True internal velocity though real pores
${v}_{s}=u/\varnothing$: Superficial *v* along straight path
$$\epsilon$$: Wall roughness
$$\xi$$: Friction ratio; $$\xi =\frac{{f}_{v} R{e}_{v}}{64}$$ & $${C}_{K}=2\xi$$
$$\mu$$: Fluid viscosity
$$\rho$$: Fluid density
$$\varnothing$$: Porosity
$$\tau$$: Shear stress ($${\tau }_{lam}, {\tau }_{turb}$$)
$$C$$: Carman
$$e$$: Real ($${L}_{e}$$), effective ($${D}_{e}$$, $${v}_{e}$$, $${f}_{e}\cdot R{e}_{e}$$)
$p$: Pore, averaged pore path
$$h$$: Hydraulic
$$K$$: Kozeny
$g$: Grain or particle
$s$: Superficial (${v}_{s}=u/\mathrm{\varnothing }$), specific (${S}_{s}$), shape (${C}_{s}$)
$u$: Apparent (medium) flow velocity
$v$: Average interstitial (pore) flow velocity
