# Supplementary material for: Application of the effective diameters of porous media to the non-Darcy flow analyses
Source: Sci Rep. 2022 Mar 29;12:5321. doi: 10.1038/s41598-022-08135-x (PMC8964684; doi:10.1038/s41598-022-08135-x)
Supplement: Supplementary file 1 — Supplementary Tables. [file 41598_2022_8135_MOESM1_ESM.docx]

Application of the Effective Diameters of Porous Media to the Non-Darcy Flow Analyses

Chang-Hoon Shin^1^†

^1^ Research Institute, Korea Gas Corporation (KOGAS), Ansan, Gyeonggi-Do, 15328, S. Korea

**Supplemental Data**

**Table S1.** Key flow variables and the effective diameters resulted from each laminar PSS case

|  | ***100*** $\mu m/s$ *in the* ***X*** *flow direction (****Mono-size pack*** *cases)* | | | | | | | |
| --- | --- | --- | --- | --- | --- | --- | --- | --- |
|  | $\boldsymbol{\Delta}\boldsymbol{P}\boldsymbol{[Pa]}$ | $\boldsymbol{v [\mu m/s]}$ | $\boldsymbol{f}_{\boldsymbol{v}}$ | $\boldsymbol{Re}_{\boldsymbol{v}}$ | $\boldsymbol{T}$ | $\boldsymbol{D}_{\boldsymbol{e}}\boldsymbol{[m]}$ | $\boldsymbol{R}\boldsymbol{e}_{\boldsymbol{e}}$ | $\boldsymbol{k [Darcy]}$ |
| **(A)** | 22.2074 | 398.493 | 3.62E+03 | 2.05E-02 | 0.5508 | 6.47E-05 | 2.56E-02 | 18.0661 |
| **(B)** | 28.7519 | 419.452 | 4.01E+03 | 2.05E-02 | 0.5536 | 5.82E-05 | 2.43E-02 | 13.9539 |
| **(C)** | 37.5756 | 454.867 | 4.23E+03 | 2.11E-02 | 0.5402 | 5.36E-05 | 2.43E-02 | 10.6771 |
| **(D)** | 49.8223 | 499.664 | 4.41E+03 | 2.20E-02 | 0.5204 | 4.97E-05 | 2.47E-02 | 8.0526 |
| **(E)** | 67.0921 | 551.517 | 4.63E+03 | 2.30E-02 | 0.4990 | 4.60E-05 | 2.52E-02 | 5.9798 |

|  | ***10*** $\mu m/s$ *in the* ***X*** *flow direction (****Multi-size pack*** *cases)* | | | | | | | |
| --- | --- | --- | --- | --- | --- | --- | --- | --- |
|  | $\boldsymbol{\Delta}\boldsymbol{P}\boldsymbol{[Pa]}$ | $\boldsymbol{v [\mu m/s]}$ | $\boldsymbol{f}_{\boldsymbol{v}}$ | $\boldsymbol{Re}_{\boldsymbol{v}}$ | $\boldsymbol{T}$ | $\boldsymbol{D}_{\boldsymbol{e}}\boldsymbol{[m]}$ | $\boldsymbol{R}\boldsymbol{e}_{\boldsymbol{e}}$ | $\boldsymbol{k [Darcy]}$ |
| **(A)** | 147.0190 | 93.3905 | 1.79E+05 | 1.97E-03 | 0.4923 | 1.29E-05 | 1.20E-03 | 0.2729 |
| **(B)** | 261.2070 | 86.4421 | 3.52E+05 | 1.73E-03 | 0.6025 | 8.40E-06 | 7.22E-04 | 0.1536 |
| **(C)** | 528.5750 | 79.1512 | 8.19E+05 | 1.53E-03 | 0.7438 | 5.08E-06 | 4.00E-04 | 0.0759 |
| **(D)** | 1127.4700 | 82.0474 | 1.58E+06 | 1.54E-03 | 0.8102 | 3.40E-06 | 2.77E-04 | 0.0356 |
| **(E)** | 2735.4600 | 88.4997 | 3.23E+06 | 1.63E-03 | 0.8467 | 2.21E-06 | 1.95E-04 | 0.0147 |

|  | ***10*** $\mu m/s$ *in the* ***Y*** *flow direction (****Multi-size pack*** *cases)* | | | | | | | |
| --- | --- | --- | --- | --- | --- | --- | --- | --- |
|  | $\boldsymbol{\Delta}\boldsymbol{P}\boldsymbol{[Pa]}$ | $\boldsymbol{v [\mu m/s]}$ | $\boldsymbol{f}_{\boldsymbol{v}}$ | $\boldsymbol{Re}_{\boldsymbol{v}}$ | $\boldsymbol{T}$ | $\boldsymbol{D}_{\boldsymbol{e}}\boldsymbol{[m]}$ | $\boldsymbol{R}\boldsymbol{e}_{\boldsymbol{e}}$ | $\boldsymbol{k [Darcy]}$ |
| **(A)** | 20.1525 | 52.4994 | 7.77E+04 | 1.11E-03 | 0.8757 | 1.95E-05 | 1.02E-03 | 1.9908 |
| **(B)** | 28.7933 | 62.2668 | 7.48E+04 | 1.25E-03 | 0.8364 | 1.82E-05 | 1.13E-03 | 1.3934 |
| **(C)** | 38.0312 | 73.4887 | 6.83E+04 | 1.42E-03 | 0.8011 | 1.76E-05 | 1.29E-03 | 1.0549 |
| **(D)** | 47.9099 | 77.9493 | 7.45E+04 | 1.46E-03 | 0.8528 | 1.57E-05 | 1.21E-03 | 0.8374 |
| **(E)** | 60.0038 | 84.3609 | 7.80E+04 | 1.55E-03 | 0.8883 | 1.43E-05 | 1.20E-03 | 0.6686 |

*$\boldsymbol{v}$ is the average interstitial flow velocity calculated from the ‘length averaged streamline velocity’ of each PSS results.

**Table S2.** Summary of key flow variations for the five mono-size pack models (65 cases)

|  | **Thickest** | | | **Thick** | | | **Base** | | | **Thin** | | | **Thinnest** | | |
| --- | --- | --- | --- | --- | --- | --- | --- | --- | --- | --- | --- | --- | --- | --- | --- |
| $\boldsymbol{u}$ | $\boldsymbol{R}\boldsymbol{e}_{\boldsymbol{e}}$ | $\boldsymbol{\Delta}\boldsymbol{P}$ | $\boldsymbol{k}$ | $\boldsymbol{R}\boldsymbol{e}_{\boldsymbol{e}}$ | $\boldsymbol{\Delta}\boldsymbol{P}$ | $\boldsymbol{k}$ | $\boldsymbol{R}\boldsymbol{e}_{\boldsymbol{e}}$ | $\boldsymbol{\Delta}\boldsymbol{P}$ | $\boldsymbol{k}$ | $\boldsymbol{R}\boldsymbol{e}_{\boldsymbol{e}}$ | $\boldsymbol{\Delta}\boldsymbol{P}$ | $\boldsymbol{k}$ | $\boldsymbol{R}\boldsymbol{e}_{\boldsymbol{e}}$ | $\boldsymbol{\Delta}\boldsymbol{P}$ | $\boldsymbol{k}$ |
| **0.00001** | 0.0026 | 2.22073 | 18.06613 | 0.0024 | 2.87516 | 13.95401 | 0.0024 | 3.75752 | 10.67726 | 0.0025 | 4.98217 | 8.05272 | 0.0025 | 6.70911 | 5.97993 |
| **0.0001** | 0.0256 | 22.2074 | 18.06605 | 0.0243 | 28.7519 | 13.95386 | 0.0243 | 37.5756 | 10.67714 | 0.0247 | 49.8223 | 8.05262 | 0.0252 | 67.0921 | 5.97984 |
| **0.001** | 0.2565 | 222.101 | 18.06385 | 0.2428 | 287.557 | 13.95202 | 0.2428 | 375.81 | 10.67561 | 0.2473 | 498.303 | 8.05133 | 0.2524 | 671.04 | 5.97878 |
| **0.005** | 1.2824 | 1111.72 | 18.04411 | 1.2140 | 1439.42 | 13.93617 | 1.2141 | 1881.27 | 10.66301 | 1.2367 | 2494.65 | 8.04121 | 1.2622 | 3359.66 | 5.97084 |
| **0.01** | 2.5647 | 2229.36 | 17.99620 | 2.4281 | 2886.59 | 13.89875 | 2.4282 | 3772.82 | 10.63396 | 2.4735 | 5003.33 | 8.01866 | 2.5243 | 6738.82 | 5.95356 |
| **0.0175** | 4.4883 | 3927.26 | 17.87760 | 4.2491 | 5085.01 | 13.80725 | 4.2493 | 6646.32 | 10.56374 | 4.3286 | 8814.91 | 7.96491 | 4.4176 | 11874 | 5.91292 |
| **0.025** | 6.4118 | 5664.37 | 17.70718 | 6.0702 | 7333.93 | 13.67616 | 6.0705 | 9585.58 | 10.46363 | 6.1837 | 12714.1 | 7.88888 | 6.3108 | 17128.6 | 5.85570 |
| **0.05** | 12.8236 | 11897.3 | 16.86097 | 12.1404 | 15403.8 | 13.02276 | 12.1409 | 20132.5 | 9.96399 | 12.3674 | 26708.4 | 7.51075 | 12.6216 | 35996.6 | 5.57275 |
| **0.075** | 19.2354 | 19004.5 | 15.83309 | 18.2106 | 24620.3 | 12.22162 | 18.2114 | 32192.5 | 9.34690 | 18.5511 | 42726.6 | 7.04245 | 18.9325 | 57617 | 5.22242 |
| **0.1** | 25.6472 | 27038.8 | 14.83794 | 24.2808 | 35056.4 | 11.44442 | 24.2819 | 45872.5 | 8.74598 | 24.7349 | 60925.3 | 6.58511 | 25.2433 | 82217.9 | 4.87972 |
| **0.25** | 64.1179 | 93556.7 | 10.72077 | 60.7019 | 120882 | 8.29735 | 60.7047 | 157890 | 6.35252 | 61.8371 | 209789 | 4.78099 | 63.1082 | 283999 | 3.53170 |
| **0.5** | 128.235 | 268846 | 7.46152 | 121.403 | 341353 | 5.87661 | 121.4094 | 437651 | 4.58356 | 123.6743 | 577212 | 3.47533 | 126.2164 | 782907 | 2.56225 |
| **0.75** | 192.353 | 513866 | 5.85561 | 182.105 | 644283 | 4.67031 | 182.1141 | 809080 | 3.71904 | 185.5114 | 1060380 | 2.83766 | 189.3246 | 1440300 | 2.08915 |

* Units: $u \left[ m/s \right], \boldsymbol{\Delta}P \left[ Pa \right], k \left[ Darcy \right]$

**Table S3.** Summary of key flow variations for the multi-size pack models in the +X directional flow conditions (52 cases)

|  | **Thickest** | | | **Thick** | | | **Base** | | | **Thin** | | | **Thinnest** | | |
| --- | --- | --- | --- | --- | --- | --- | --- | --- | --- | --- | --- | --- | --- | --- | --- |
| $\boldsymbol{u}$ | $\boldsymbol{R}\boldsymbol{e}_{\boldsymbol{e}}$ | $\boldsymbol{\Delta}\boldsymbol{P}$ | $\boldsymbol{k}$ | $\boldsymbol{R}\boldsymbol{e}_{\boldsymbol{e}}$ | $\boldsymbol{\Delta}\boldsymbol{P}$ | $\boldsymbol{k}$ | $\boldsymbol{R}\boldsymbol{e}_{\boldsymbol{e}}$ | $\boldsymbol{\Delta}\boldsymbol{P}$ | $\boldsymbol{k}$ | $\boldsymbol{R}\boldsymbol{e}_{\boldsymbol{e}}$ | $\boldsymbol{\Delta}\boldsymbol{P}$ | $\boldsymbol{k}$ | $\boldsymbol{R}\boldsymbol{e}_{\boldsymbol{e}}$ | $\boldsymbol{\Delta}\boldsymbol{P}$ | $\boldsymbol{k}$ |
| **0.000001** | 0.0001 | 14.7018 | 0.27289 | 0.0001 | 26.1203 | 0.15360 | 0.0000 | 52.8563 | 0.07590 | 0.0000 | 112.742 | 0.03559 | 0.0000 | 273.535 | 0.01467 |
| **0.00001** | 0.0012 | 147.019 | 0.27289 | 0.0007 | 261.207 | 0.15359 | 0.0004 | 528.575 | 0.07590 | 0.0003 | 1127.47 | 0.03558 | 0.0002 | 2735.46 | 0.01467 |
| **0.0001** | 0.0120 | 1470.3 | 0.27287 | 0.0072 | 2612.48 | 0.15357 | 0.0040 | 5287.03 | 0.07588 | 0.0028 | 11279.4 | 0.03557 | 0.0020 | 27366.1 | 0.01466 |
| **0.001** | 0.1196 | 14713.9 | 0.27267 | 0.0722 | 26166.2 | 0.15333 | 0.0400 | 52999 | 0.07570 | 0.0277 | 113271 | 0.03542 | 0.0195 | 274818 | 0.01460 |
| **0.005** | 0.5981 | 73840.4 | 0.27167 | 0.3612 | 131795 | 0.15221 | 0.2002 | 267978 | 0.07486 | 0.1386 | 577453 | 0.03474 | 0.0975 | 1399790 | 0.01433 |
| **0.01** | 1.1963 | 148476 | 0.27021 | 0.7224 | 266165 | 0.15073 | 0.4005 | 543742 | 0.07378 | 0.2773 | 1182470 | 0.03393 | 0.1951 | 2858740 | 0.01403 |
| **0.025** | 2.9907 | 378889 | 0.26472 | 1.8061 | 686567 | 0.14609 | 1.0012 | 1419020 | 0.07068 | 0.6932 | 3131160 | 0.03203 | 0.4877 | 7614630 | 0.01317 |
| **0.05** | 5.9814 | 793333 | 0.25286 | 3.6122 | 1452590 | 0.13810 | 2.0024 | 3040880 | 0.06597 | 1.3864 | 6766210 | 0.02965 | 0.9754 | 16928900 | 0.01185 |
| **0.075** | 8.9721 | 1256600 | 0.23946 | 5.4184 | 2310490 | 0.13023 | 3.0036 | 4877510 | 0.06169 | N/A | N/A | N/A | N/A | N/A | N/A |
| **0.1** | 11.9628 | 1772770 | 0.22631 | 7.2245 | 3263940 | 0.12292 | 4.0048 | 6937500 | 0.05783 | N/A | N/A | N/A | N/A | N/A | N/A |
| **0.15** | 17.9441 | 2956940 | 0.20352 | 10.8367 | 5450820 | 0.11041 | 6.0071 | 11712900 | 0.05138 | N/A | N/A | N/A | N/A | N/A | N/A |
| **0.25** | 29.9069 | 5857920 | 0.17122 | 18.0612 | 10862500 | 0.09234 | 10.0119 | 23520600 | 0.04264 | N/A | N/A | N/A | N/A | N/A | N/A |

**Table S4.** Summary of key flow variations for the multi-size pack models in the +Y directional flow conditions (65 cases)

|  | **Thickest** | | | **Thick** | | | **Base** | | | **Thin** | | | **Thinnest** | | |
| --- | --- | --- | --- | --- | --- | --- | --- | --- | --- | --- | --- | --- | --- | --- | --- |
| $\boldsymbol{u}$ | $\boldsymbol{R}\boldsymbol{e}_{\boldsymbol{e}}$ | $\boldsymbol{\Delta}\boldsymbol{P}$ | $\boldsymbol{k}$ | $\boldsymbol{R}\boldsymbol{e}_{\boldsymbol{e}}$ | $\boldsymbol{\Delta}\boldsymbol{P}$ | $\boldsymbol{k}$ | $\boldsymbol{R}\boldsymbol{e}_{\boldsymbol{e}}$ | $\boldsymbol{\Delta}\boldsymbol{P}$ | $\boldsymbol{k}$ | $\boldsymbol{R}\boldsymbol{e}_{\boldsymbol{e}}$ | $\boldsymbol{\Delta}\boldsymbol{P}$ | $\boldsymbol{k}$ | $\boldsymbol{R}\boldsymbol{e}_{\boldsymbol{e}}$ | $\boldsymbol{\Delta}\boldsymbol{P}$ | $\boldsymbol{k}$ |
| **0.000001** | 0.0001 | 2.01525 | 1.990820 | 0.0001 | 2.87932 | 1.393385 | 0.0001 | 3.80311 | 1.054926 | 0.0001 | 4.79097 | 0.837409 | 0.0001 | 6.00036 | 0.668627 |
| **0.00001** | 0.0010 | 20.1525 | 1.990820 | 0.0011 | 28.7933 | 1.393380 | 0.0013 | 38.0312 | 1.054923 | 0.0012 | 47.9099 | 0.837405 | 0.0012 | 60.0038 | 0.668624 |
| **0.0001** | 0.0102 | 201.528 | 1.990790 | 0.0113 | 287.938 | 1.393356 | 0.0129 | 380.322 | 1.054896 | 0.0121 | 479.115 | 0.837377 | 0.0120 | 600.064 | 0.668595 |
| **0.001** | 0.1021 | 2015.57 | 1.990504 | 0.1129 | 2879.98 | 1.393065 | 0.1287 | 3804.26 | 1.054607 | 0.1214 | 4792.79 | 0.837091 | 0.1197 | 6003.16 | 0.668315 |
| **0.005** | 0.5105 | 10085 | 1.989093 | 0.5645 | 14414.1 | 1.391693 | 0.6435 | 19045.9 | 1.053245 | 0.6071 | 24002.5 | 0.835746 | 0.5984 | 30074.7 | 0.667006 |
| **0.01** | 1.0211 | 20190.7 | 1.987053 | 1.1291 | 28868.6 | 1.389745 | 1.2870 | 38160.1 | 1.051360 | 1.2141 | 48110.3 | 0.833917 | 1.1968 | 60309.1 | 0.665240 |
| **0.025** | 2.5527 | 50678.9 | 1.979127 | 2.8226 | 72548.5 | 1.382523 | 3.2176 | 96017.8 | 1.044598 | 3.0353 | 121200 | 0.827558 | 2.9920 | 152145 | 0.659240 |
| **0.05** | 5.1054 | 102294 | 1.961014 | 5.6453 | 146766 | 1.366802 | 6.4351 | 194666 | 1.030483 | 6.0705 | 246180 | 0.814851 | 5.9840 | 309712 | 0.647699 |
| **0.075** | 7.6581 | 155223 | 1.938501 | 8.4679 | 223215 | 1.348028 | 9.6527 | 296717 | 1.014098 | 9.1058 | 375841 | 0.800605 | 8.9760 | 473701 | 0.635211 |
| **0.1** | 10.2107 | 209707 | 1.913145 | 11.2906 | 302226 | 1.327483 | 12.8702 | 402628 | 0.996453 | 12.1410 | 510711 | 0.785571 | 11.9679 | 644650 | 0.622353 |
| **0.25** | 25.5268 | 574656 | 1.745392 | 28.2264 | 837041 | 1.198269 | 32.1756 | 1129720 | 0.887831 | 30.3525 | 1442940 | 0.695109 | 29.9199 | 1830320 | 0.547992 |
| **0.375** | 38.2903 | 929439 | 1.618718 | 42.3396 | 1361190 | 1.105283 | 48.2634 | 1852070 | 0.812334 | 45.5288 | 2376820 | 0.632989 | 44.8798 | 3021500 | 0.497931 |
| **0.5** | 51.0537 | 1326650 | 1.512079 | 56.4528 | 1947690 | 1.029938 | 64.3512 | 2665180 | 0.752670 | 60.7050 | 3434670 | 0.584044 | 59.8397 | 4377680 | 0.458234 |

**Table S5.** Total pressure drops [Pa] and errors [%] using Equations (6) and (8), respectively for the (a) Mono-size pack, (b) Multi-size pack Y- and (c) Multi-size pack X-directional cases

| **(a) Mono-size** | **Thickest** | | **Thick** | | **Base** | | **Thin** | | **Thinnest** | |
| --- | --- | --- | --- | --- | --- | --- | --- | --- | --- | --- |
| **0.00001** | 2.221E+00 | (0.00%) | 2.875E+00 | (0.00%) | 3.758E+00 | (0.00%) | 4.982E+00 | (0.00%) | 6.709E+00 | (0.00%) |
| **0.0001** | 2.221E+01 | (0.00%) | 2.875E+01 | (0.00%) | 3.758E+01 | (0.00%) | 4.982E+01 | (0.00%) | 6.709E+01 | (0.00%) |
| **0.001** | 2.222E+02 | (0.05%) | 2.877E+02 | (0.04%) | 3.760E+02 | (0.04%) | 4.985E+02 | (0.05%) | 6.714E+02 | (0.05%) |
| **0.005** | 1.117E+03 | (0.50%) | 1.446E+03 | (0.47%) | 1.890E+03 | (0.48%) | 2.508E+03 | (0.52%) | 3.378E+03 | (0.56%) |
| **0.01** | 2.255E+03 | (1.17%) | 2.919E+03 | (1.11%) | 3.817E+03 | (1.16%) | 5.066E+03 | (1.25%) | 6.831E+03 | (1.37%) |
| **0.0175** | 4.007E+03 | (2.03%) | 5.185E+03 | (1.96%) | 6.784E+03 | (2.07%) | 9.015E+03 | (2.27%) | 1.217E+04 | (2.51%) |
| **0.025** | 5.813E+03 | (2.62%) | 7.520E+03 | (2.54%) | 9.848E+03 | (2.73%) | 1.310E+04 | (3.03%) | 1.771E+04 | (3.40%) |
| **0.05** | 1.223E+04 | (2.81%) | 1.582E+04 | (2.70%) | 2.076E+04 | (3.12%) | 2.771E+04 | (3.77%) | 3.763E+04 | (4.55%) |
| **0.075** | 1.926E+04 | (1.36%) | 2.491E+04 | (1.17%) | 3.276E+04 | (1.75%) | 4.387E+04 | (2.67%) | 5.980E+04 | (3.79%) |
| **0.1** | 2.691E+04 | (0.48%) | 3.479E+04 | (0.76%) | 4.583E+04 | (0.08%) | 6.156E+04 | (1.04%) | 8.422E+04 | (2.43%) |
| **0.25** | 8.567E+04 | (8.43%) | 1.107E+05 | (8.44%) | 1.471E+05 | (6.86%) | 2.001E+05 | (4.63%) | 2.779E+05 | (2.13%) |
| **0.5** | 2.327E+05 | (13.44%) | 3.005E+05 | (11.97%) | 4.025E+05 | (8.04%) | 5.542E+05 | (3.98%) | 7.808E+05 | (0.27%) |
| **0.75** | 4.412E+05 | (14.15%) | 5.694E+05 | (11.62%) | 7.663E+05 | (5.29%) | 1.062E+06 | (0.20%) | 1.508E+06 | (4.74%) |

| **(b) Multi-size (X)** | **Thickest** | | **Thick** | | **Base** | | **Thin** | | **Thinnest** | |
| --- | --- | --- | --- | --- | --- | --- | --- | --- | --- | --- |
| **0.000001** | 1.470E+01 | (0.00%) | 2.612E+01 | (0.00%) | 5.286E+01 | (0.00%) | 1.127E+02 | (0.00%) | 2.735E+02 | (0.00%) |
| **0.00001** | 1.470E+02 | (0.00%) | 2.612E+02 | (0.00%) | 5.286E+02 | (0.00%) | 1.127E+03 | (0.00%) | 2.735E+03 | (0.00%) |
| **0.0001** | 1.470E+03 | (0.01%) | 2.612E+03 | (0.02%) | 5.286E+03 | (0.02%) | 1.127E+04 | (0.04%) | 2.735E+04 | (0.04%) |
| **0.001** | 1.471E+04 | (0.05%) | 2.612E+04 | (0.16%) | 5.286E+04 | (0.26%) | 1.128E+05 | (0.46%) | 2.736E+05 | (0.46%) |
| **0.005** | 7.380E+04 | (0.06%) | 1.309E+05 | (0.69%) | 2.646E+05 | (1.27%) | 5.641E+05 | (2.30%) | 1.368E+06 | (2.24%) |
| **0.01** | 1.488E+05 | (0.19%) | 2.630E+05 | (1.18%) | 5.305E+05 | (2.43%) | 1.131E+06 | (4.39%) | 2.741E+06 | (4.11%) |
| **0.025** | 3.845E+05 | (1.47%) | 6.741E+05 | (1.82%) | 1.349E+06 | (4.95%) | 2.870E+06 | (8.35%) | 6.962E+06 | (8.57%) |
| **0.05** | 8.194E+05 | (3.28%) | 1.427E+06 | (1.79%) | 2.841E+06 | (6.56%) | 6.128E+06 | (9.43%) | 1.559E+07 | (7.90%) |
| **0.075** | 1.309E+06 | (4.20%) | 2.276E+06 | (1.50%) | 4.572E+06 | (6.27%) | 1.035E+07 | N/A | 3.415E+07 | N/A |
| **0.1** | 1.855E+06 | (4.65%) | 3.227E+06 | (1.13%) | 6.593E+06 | (4.96%) | 1.619E+07 | N/A | 1.078E+08 | N/A |
| **0.15** | 3.115E+06 | (5.34%) | 5.441E+06 | (0.18%) | 1.162E+07 | (0.80%) | 3.555E+07 | N/A | 2.884E+10 | N/A |
| **0.25** | 6.308E+06 | (7.68%) | 1.113E+07 | (2.43%) | 2.593E+07 | (10.26%) | 1.227E+08 | N/A | 9.722E+08 | N/A |

| **(c) Multi-size (Y)** | **Thickest** | | **Thick** | | **Base** | | **Thin** | | **Thinnest** | |
| --- | --- | --- | --- | --- | --- | --- | --- | --- | --- | --- |
| **0.000001** | 2.015E+00 | (0.00%) | 2.879E+00 | (0.00%) | 3.803E+00 | (0.00%) | 4.791E+00 | (0.00%) | 6.000E+00 | (0.00%) |
| **0.00001** | 2.015E+01 | (0.00%) | 2.879E+01 | (0.00%) | 3.803E+01 | (0.00%) | 4.791E+01 | (0.00%) | 6.000E+01 | (0.00%) |
| **0.0001** | 2.015E+02 | (0.00%) | 2.879E+02 | (0.00%) | 3.803E+02 | (0.00%) | 4.791E+02 | (0.00%) | 6.000E+02 | (0.00%) |
| **0.001** | 2.016E+03 | (0.00%) | 2.880E+03 | (0.00%) | 3.804E+03 | (0.01%) | 4.792E+03 | (0.02%) | 6.002E+03 | (0.03%) |
| **0.005** | 1.009E+04 | (0.07%) | 1.442E+04 | (0.07%) | 1.906E+04 | (0.08%) | 2.401E+04 | (0.03%) | 3.007E+04 | (0.01%) |
| **0.01** | 2.024E+04 | (0.24%) | 2.894E+04 | (0.26%) | 3.827E+04 | (0.29%) | 4.820E+04 | (0.19%) | 6.038E+04 | (0.12%) |
| **0.025** | 5.113E+04 | (0.89%) | 7.324E+04 | (0.96%) | 9.708E+04 | (1.11%) | 1.223E+05 | (0.87%) | 1.532E+05 | (0.70%) |
| **0.05** | 1.042E+05 | (1.89%) | 1.498E+05 | (2.06%) | 1.993E+05 | (2.38%) | 2.510E+05 | (1.94%) | 3.148E+05 | (1.66%) |
| **0.075** | 1.594E+05 | (2.70%) | 2.298E+05 | (2.95%) | 3.069E+05 | (3.42%) | 3.864E+05 | (2.82%) | 4.853E+05 | (2.45%) |
| **0.1** | 2.167E+05 | (3.33%) | 3.133E+05 | (3.66%) | 4.198E+05 | (4.26%) | 5.287E+05 | (3.52%) | 6.647E+05 | (3.10%) |
| **0.25** | 6.045E+05 | (5.19%) | 8.874E+05 | (6.01%) | 1.210E+06 | (7.13%) | 1.525E+06 | (5.69%) | 1.928E+06 | (5.32%) |
| **0.375** | 9.855E+05 | (6.03%) | 1.462E+06 | (7.38%) | 2.017E+06 | (8.90%) | 2.543E+06 | (6.98%) | 3.225E+06 | (6.74%) |
| **0.5** | 1.419E+06 | (6.96%) | 2.123E+06 | (9.01%) | 2.958E+06 | (10.98%) | 3.730E+06 | (8.61%) | 4.745E+06 | (8.39%) |
